# GSK-3 Inhibition Is Cytotoxic in Glioma Stem Cells through Centrosome Destabilization and Enhances the Effect of Radiotherapy in Orthotopic Models

**DOI:** 10.3390/cancers13235939

**Published:** 2021-11-25

**Authors:** Anke Brüning-Richardson, Gary C. Shaw, Daniel Tams, Tim Brend, Hitesh Sanganee, Simon T. Barry, Gregory Hamm, Richard J. A. Goodwin, John G. Swales, Henry King, Lynette Steele, Ruth Morton, Anastasia Widyadari, Thomas A. Ward, Filomena Esteves, Marjorie Boissinot, Georgia Mavria, Alastair Droop, Sean E. Lawler, Susan C. Short

**Affiliations:** 1 Leeds Institute of Medical Research at St James’s, University of Leeds, Leeds LS9 7TF, UK; G.C.Shaw@leeds.ac.uk (G.C.S.); D.Tams@leeds.ac.uk (D.T.); T.Brend@leeds.ac.uk (T.B.); H.O.King@leeds.ac.uk (H.K.); L.Steele@leeds.ac.uk (L.S.); R.Morton@leeds.ac.uk (R.M.); A.Widyadari1@leeds.ac.uk (A.W.); Thomas.Ward@md.catapult.org.uk (T.A.W.); F.E.O.Esteves@leeds.ac.uk (F.E.); M.Boissinot@leeds.ac.uk (M.B.); G.Mavria@leeds.ac.uk (G.M.); 2 Discovery Sciences BioPharmaceuticals R&D, AstraZeneca, Cambridge CB2 8PA, UK; Sanganee_H@astrazeneca.com; 3 Bioscience, Early Oncology, Oncology R&D, AstraZeneca, Cambridge CB2 8PA, UK; Simon.T.Barry@astrazeneca.com; 4 Imaging and Data Analytics, Clinical Pharmacology and Safety Sciences, BioPharmaceuticals R&D, AstraZeneca, Cambridge CB2 8PA, UK; Gregory.Hamm@astrazeneca.com (G.H.); Richard.Goodwin@astrazeneca.com (R.J.A.G.); John.Swales@astrazeneca.com (J.G.S.); 5 Leeds MRC Medical Bioinformatics Centre, University of Leeds, Leeds LS9 7TF, UK; ad33@sanger.ac.uk; 6 Pathology & Laboratory Medicine, Brown University Cancer Center, Brown University, Providence, RI 02903, USA; Sean_Lawler@brown.edu

**Keywords:** glioblastoma, GSK-3, centrosome, radiation, cytotoxicity

## Abstract

**Simple Summary:**

High-grade gliomas remain difficult-to-treat cancers. Novel treatment options include targeting glycogen synthase kinase 3 (GSK-3) to induce cell death but the mode of drug activity remains unknown and combination with conventional treatment including radiotherapy has not been explored. Here, we describe the effect of targeting GSK-3 with the inhibitor AZD2858 in in vitro and in vivo models of glioma. We established that AZD2858 exposure induces mitotic defects leading to cell death in patient-derived glioma cell lines and tumor growth delay in glioma xenografts. Co-administration also enhanced the effect of radiotherapy. We therefore propose AZD2858 as an adjuvant to radiotherapy in high-grade glioma.

**Abstract:**

Background: Previous data on glycogen synthase kinase 3 (GSK-3) inhibition in cancer models support a cytotoxic effect with selectivity for tumor cells compared to normal tissue but the effect of these inhibitors in glioma has not been widely studied. Here, we investigate their potential as cytotoxics in glioma. Methods: We assessed the effect of pharmacologic GSK-3 inhibition on established (U87, U251) and patient-derived (GBM1, GBM4) glioblastoma (GBM) cell lines using cytotoxicity assays as well as undertaking a detailed investigation of the effect on cell cycle, mitosis, and centrosome biology. We also assessed drug uptake and efficacy of GSK-3 inhibition alone and in combination with radiation in xenograft models. Results: Using the selective GSK-3 inhibitor AZD2858, we demonstrated single agent cytotoxicity in two patient-derived glioma cell lines (GBM1, GBM4) and two established cell lines (U251 and U87) with IC_50_ in the low micromolar range promoting centrosome disruption, failed mitosis, and S-phase arrest. Glioma xenografts exposed to AZD2858 also showed growth delay compared to untreated controls. Combined treatment with radiation increased the cytotoxic effect of clinical radiation doses in vitro and in orthotopic glioma xenografts. Conclusions: These data suggest that GSK-3 inhibition promotes cell death in glioma through disrupting centrosome function and promoting mitotic failure and that AZD2858 is an effective adjuvant to radiation at clinical doses.

## 1. Introduction

Malignant gliomas remain amongst the most lethal of cancers and its commonest type, glioblastoma (GBM), is associated with survival of little over a year despite combination treatment of maximal surgery, high dose radiotherapy, and chemotherapy. The main challenge in this disease is local control since metastasis outside the CNS is very rare. The underlying mechanisms of treatment resistance are still poorly understood but include upregulation of DNA damage response in a subpopulation of stem-like cells, which drives chemo and radioresistance as well as a highly migratory phenotype, which limits efficacy of local treatments including surgery [1,2,3,4]. Definition of novel targeting strategies to alter this treatment-resistant phenotype is a major unmet need in neuro-oncology. The glycogen synthase kinase 3 (GSK-3) signaling network is of interest in this regard as it has established roles in controlling neuronal differentiation, survival, and motility. Two isoforms of GSK-3 exist, GSK-3 alpha and GSK-3 beta, which are structurally similar with 67% homology in amino acid content and a single amino acid change in the ATP binding domain [5]. Both isoforms have distinct but overlapping functions and are inhibited by GSK-3 inhibitors. Development in the field has been limited by lack of specific inhibitors, although the development of a tool compound with selectivity has been reported [6].

GSK-3 beta has been previously identified as a potential therapeutic target in several difficult to treat cancers including pancreatic cancer and GBM [7,8,9]. It is known to have tissue specific functions and is particularly abundant in the brain during development with a pronounced neuronal localization. In a neural context, activation of GSK-3 beta has anti-apoptotic, pro-proliferative, and pro-migratory effects, hence overlapping with the phenotype of treatment-resistant glial tumor cells. Additionally, key characteristics of the specific stem cell-like subpopulation, including neurosphere formation, may be maintained through regulation of GSK-3/Wnt/beta-catenin signaling [10,11,12] and GSK-3 may also mediate epithelial-to-mesenchymal transition in GBM cells [13]. Primary GBM are characterized by GSK-3 beta expression, and high levels of total and phosphorylated protein have been reported in GBM compared with normal brain, indicating an association with the malignant phenotype [14]. Recently, GSK-3 inhibition has been shown to induce apoptosis and mitotic catastrophe through centrosomal dysregulation in several cancer types, but the underlying mechanism remains unknown [6,15,16]. We aimed to characterize cytotoxicity induced by GSK-3 inhibition with the potent and selective GSK-3 inhibitor AZD2858 in established and patient-derived glioma cell lines. Our data utilizing in vitro and in vivo models confirm GSK-3 as a candidate target for treatment of malignant glioma; we demonstrate a cytotoxic mechanism associated with centrosome destabilization and suggest a potential role as an adjuvant to radiotherapy.

## 2. Materials and Methods

### 2.1. Cell Lines

U87 and U251 adult glioma cell lines were obtained from ATCC (Manassas, VA, USA). The patient-derived tumour models GBM1, GBM4, and the adult human brain progenitor line NP1 were a kind gift from H. Wurdak (Stem Cell and Brain Tumour Group, University of Leeds, Leeds, UK) and were used as previously described in [17,18].

The identity of all cell lines was verified by serial tandem repeat profiling through in-house cell line authentication services and were negative for mycoplasma contamination.

Ethical approval for the use of patient-derived cell lines was granted through the Leeds Research Ethics Committee (reference number 10-H1306-7).

### 2.2. In Vitro Culture

U87 and U251 cell lines were grown in DMEM (Sigma) supplemented with 10% fetal calf serum (Sigma, Poole, UK) and penicillin/streptomycin as previously described [17,18,19]. For the patient-derived cell lines, specific growth media promoting the maintenance of stemness was used as described previously. Briefly, brain tumor-initiating neurobasal medium (ThermoFisher Scientific, Waltham, MA, USA supplemented with N2, B27, human recombinant bFGF, and EGF was used to grow cells in cell culture plastics coated with poly-l-ornithine and laminin.

NP1 and NHA (ScienCell Research Laboratories; kindly gifted by Dr Heiko Wurdak) were grown as previously described [17,18].

### 2.3. AZD2858 Preparation

AZD2858 was obtained from AstraZeneca (Macclesfield, UK) prepared as an ethanesulfonic acid salt. The molecular weight is 673.79 and the Parent molecular weight (no salt) is 453.53. The compound and its potency and selectivity has been previously described [20]. AZD2858 was resuspended in DMSO and stored at −20 °C at a concentration of 10 mM; for in vitro studies, all dilutions were made in growth medium, and for in vivo studies, dilutions were made in Milli-Q water.

### 2.4. 3D Spheroid Generation

Spheroids were generated from established and patient-derived cell lines as previously described [21]. Cells were seeded at 1 × 10^3^/well in a low adherence 96-well plate (Corning, Liversedge, UK) and allowed to form spheroids over 72 h. For the spheroid assay, the growth medium was replaced with collagen (Collagen type 1 rat tail, Corning, Liversedge, UK). Collagen polymerization was initiated by the addition of 1 M NaOH. Invasion into the collagen was observed over 72 h post collagen embedding. After 72 h, the spheroids were prepared for immunohistochemistry as previously described [21]. Individual spheroids contained within collagen plugs were sectioned and hematoxylin and eosin (H&E) stained according to Cheng et al. [19].

### 2.5. Cytotoxicity Assays

MTT (3-(4,5-dimethylthiazol-2-yl)-2,5-diphenyltetrazolium bromide) assays were carried out to assess the effect of AZD2858 on cell viability as previously described in Levesley et al. [22]. Briefly, cells were seeded at 2.5 × 10^3^/well. AZD2858 was added in triplicate in 10-fold dilutions (0.01–10 mM) in DMSO. Control wells containing DMSO only were also included. After 72 h incubation, MTT was added to the wells for 3 h. Purple formazan crystals were solubilized with DMSO. Optical density read outs at 540 nm were obtained on an ELISA plate reader. The MTT assays were repeated three times for each cell line.

### 2.6. Live Cell Imaging

To investigate the effect of AZD2858 on cell viability/mitosis, cells were seeded at subconfluence in 96-well flat bottom plates and treated with AZD2858 concentrations between 0.01 and 20 µM or mock-treated with a DMSO control. Cell death or mitotic events were recorded by live cell imaging with the Incucyte imaging system (Essen BioScience, Newark, UK) over 72 h or over 1 week at 30 min intervals. AVI movies were generated and analyzed using ImageJ as previously described [22]. At least 50 mitotic cells/treatment were recorded to determine duration of mitosis, completion of mitosis, cytokinetic failure, and cell death during mitosis.

### 2.7. Immunofluorescence Microscopy

For immunofluorescence, cells were grown on sterile coverslips in six-well plates. AZD2858 was added at predetermined concentrations and the cells were allowed to incubate for 2 h or 72 h depending on the experimental set-up. Cells were then fixed with 4% paraformaldehyde or with 100% methanol as previously described [21]. For PFA fixation, cells were repermeabilized with 0.1% Triton-X 100 (Sigma, Poole, UK) prior to blocking with 0.1% skimmed milk powder. Primary and secondary antibodies in the same blocking solution were applied for one hour. Cells were mounted in Fluoromount-G (ThermoFisher Scientific, Waltham, MA, USA and images generated by confocal microscopy using an A1R Eclipse Ti-E LSM Confocal Microscope (Nikon Instruments, Surbiton, UK).

Primary antibodies used included rat anti-tubulin (1/500, #MCA77G, Bio-Rad, Hercules, CA, USA), mouse anti-acetylated tubulin (1/500, clone 6-11B-1, #T7451, Sigma, Dorset, UK), rabbit anti-pericentrin (#ABT59, Sigma, Poole, UK), and rabbit anti-beta-catenin (#ab6302, Abcam, Cambridge, UK). Cells were also stained with 4′,6-Diamidino-2-Phenylindole (DAPI) for nuclear localization as per manufacturers’ instructions and with phalloidin (Molecular Probes, ThermoFisher Scientific, Altrincham, UK) as a marker of the actin cytoskeleton.

### 2.8. Cell Cycle Analysis

Cells were mock-treated or treated with AZD2858 at 5-fold dilutions from 50 to 0.0032 µM and prepared for FACS analysis as previously described [22] in FACS buffer (PBS and 0.1% *w*/*v* BSA and 0.1% *v*/*v* Tween-20) with 20 g/mL propidium iodide (PI) and 200 µg RNase A. The stage of the cell cycle was determined in the whole cell population using flow cytometry imaging of the PI stained and fixed cells. Specific algorithms in ModFit LT 3.2 (Verity software for ploidy and cell cycle analysis) automatically determined stage of the cell cycle based on DNA content. The experiments were repeated three times for each cell line.

### 2.9. Western Blot

Cell lysates were generated from cells mock-treated or treated with AZD2858 at 0, 0.1, 1, and 10 µM. The lysates were prepared for SDS-PAGE electrophoresis as previously described [22]. Separated proteins were probed with the following antibodies in Western blots as previously described [22]. Antibodies used included anti-beta-catenin (1/1000, #9562, CST, Cambridge, UK), phospho-beta-catenin (1/1000, #9561, CST, Cambridge, UK), GSK-3 alpha (1/1000, #9338, CST, Cambridge, UK), GSK-3 beta (1/1000, #9315, CST, Cambridge, UK), TACC3 (1/500, #ab56595, Abcam, Cambridge, UK), Akt (1/1000, #9272, CST), phospho-Akt (1/1000, #9271, CST, Cambridge, UK), and beta-actin (1/10,000, #A1978, Sigma, Poole, UK).

### 2.10. Real-Time PCR

RNA was isolated using the Qiagen mini kit including a DNase step. cDNA was made using the Applied Biosystems (Warrington, UK) high capacity reverse transcription kit. qPCR was carried out with TaqMan primer probe sets from Applied Biosystems (Warrington, UK) (GAPDH and Cyclin D) and TaqMan Master Mix. This was for 40 cycles with amplification taking place in Applied Biosystems real-time PCR reaction plates. All reactions were carried out according to manufacturers’ instructions.

### 2.11. Immunohistochemistry

For immunohistochemistry, a protocol as previously described was followed [19]. Briefly, all slides were dewaxed and hydrated. Antigen retrieval was achieved with a pressure cooker using Tris EDTA buffer pH 9.0. For mouse antibodies, the incubation time was 1 h (SOX2 1/250, clone #2456120, R&D, Abingdon, UK; anti-human Nuclei 1/400, #MAB4383, Merck Millipore, Watford, UK) Cyclin D1 (1/50, clone #P2DIIFII, Novocastra, Newcastle upon Tyne, UK). A Mouse on Mouse Polymer IHC Kit (Abcam, Cambridge, UK) was used according to the manufacturer’s instructions for staining. For all rabbit antibodies (Ki67, 1/200, clone SP6 #RM-9106-R7, (ThermoFisher Scientific, Altham, UK); cleaved caspase 3, 1/500, clone 5A1E, #9664 (CST, Cambridge, UK); beta-catenin, 1/300, #9562 (CST, Cambridge, UK) after incubation with the primary antibodies for 1 h, slides were washed and a secondary anti-rabbit polymer HRP from MenaPath (#MP-XCP-P0100, A.Menarini Diagnostics, Wokingham, UK) was used according to manufacturer’s instruction, as for the mouse antibodies. 3,3′diaminobenzidine (DAB) was used as chromogen substrate (ImmPACT DAB, #SK-4105, Vector Laboratories, Cambridge, UK). Nuclei were counterstained with Mayer’s hematoxylin.

### 2.12. Microarray Data

Cells from three different cell lines (U251, GBM1, and GBM4) were seeded at 0.5 × 10^6^ cells/10 cm^2^ Petri dish (laminin coated for patient-derived cell lines) and allowed to attach overnight. The following day, cells were treated with 1 µM AZD2858 or mock-treated with 0.01% DMSO in fresh media. At 8 h and 24 h intervals, cells were harvested and snap frozen on dry ice (in triplicate). Cell pellets were processed for RNA isolation and QC for microarray data generation. Twenty-four samples (2 replicates of 3 cell lines, 2 timepoints, 2 treatments) were run on Agilent SurePrint G3 Human Gene Expression 8 × 60 K v2 arrays. Six arrays were used on each of two slides (using two dyes). The slides were processed and analyzed in-house (Leeds MRC Medical Bioinformatics Centre). Microarray data were processed in R using the LIMMA [23] package as follows: First, per-array correction was performed using first a normal and exponential convolution method (“normexp”) followed by LOESS normalization to normalize the multiple subarrays on each chip [24]. Multiple arrays were normalized using quantile normalization across the average intensity values (“Aquantile”) [25,26]. Differential gene analysis was also performed using LIMMA. Results were computed using a 2-colour linear model followed by empirical Bayes moderation of the standard errors. Multiple testing correction was performed using Benjamini and Hochberg false discovery rate (FDR) adjustment. An FDR threshold of 0.005 was applied to select only highly significant genes. In the generated data, treatment versus non-treated was compared, taking into account cell line and FDR threshold, 0.005. From these results, all probes were pulled out that were annotated as GO:0000278 ‘mitotic cell cycle’ or more specific. The sorted, annotated data for the genes that were annotated with any of these terms were given in lemma-results.cvs. From these results, the genes with the highest significant values (*p*-values) and known role in mitosis were noted, which were also associated with the observed phenotypes.

### 2.13. AZD2858 Drug Penetration Studies

Analytical-grade acetonitrile, methanol, and trifluoroacetic acid were obtained from FisherScientific (Loughborough, Leicestershire, UK). (Hydroxypropyl)-methylcellulose and polyvinylpyrrolidone were obtained from Sigma-Aldrich (Poole,, UK). Isopropanol and isopentane were purchased from VWR International (Lutterworth, Leicestershire, UK).

#### 2.13.1. Tissue Embedding

Whole mouse brain tissue from xenograft experiments with orthotopic U87 was embedded in (Hydroxypropyl)-methylcellulose (7.5% *w*/*v*) modified with polyvinylpyrrolidone (2.5% *w*/*v*). The embedding media was pre-conditioned on ice for 30 min prior to addition of the tissue to minimize any increase in temperature. The resulting block was then immediately snap frozen by submersion in dry ice-cooled isopropanol for several minutes and subsequently submerged in dry ice-cooled isopentane for 1–2 min. The resulting frozen tissue blocks were transferred to −80 °C storage prior to tissue sectioning.

#### 2.13.2. Tissue Sectioning

Embedded tissues were cryosectioned on a CM3050S cryomicrotome (Leica Biosystems, Nussloch, Germany) at a thickness of 10 μm and thaw-mounted onto indium tin oxide (ITO)-coated MALDI target slides (Bruker Daltonics, Bremen, Germany). Thaw-mounted slides were immediately desiccated using a stream of dry N2 prior to vacuum packing and storage at −80 °C until analysis.

#### 2.13.3. MALDI Matrix Application

Vacuum packed, thaw-mounted tissue sections were allowed to reach room temperature after removal from −80 °C storage, prior to breaking the vacuum seal. Optical images were taken using a standard flat-bed scanner (Seiko Epson, Nagano, Japan) prior to MALDI matrix application. Matrix coating was applied using a TM-Sprayer (HTX technologies, Chapel Hill, NC, USA) set at 75 °C and performing 8 passes, with a back-up flow of 50% methanol/water *v*/*v* at a flow rate of 0.08 mL/min and nebulized with nitrogen at 8 psi. The matrix used in the experiments was 2,5-Dihydroxybenzoic acid at a concentration of 35 mg/mL in acetonitrile/water/trifluoroacetic acid (50/50/0.1% *v*/*v*/*v*).

#### 2.13.4. MALDI Mass Spectrometry Imaging (MSI)

MALDI-MSI experiments were carried out in positive ion reflectron mode over a mass range of m/z 100 to 1000 using a MALDI rapifleX Tissuetyper MALDITOF/TOF MS (Bruker Daltonics, Bremen, Germany) equipped with a 10 kHz Smartbeam 3DTM Nd:YAG laser. Data collected on the rapifleX was at a spatial resolution of 50 µm, summing up 400 laser shots/raster position. FlexImaging 5.0 (Bruker Daltonics, Bremen, Germany) software was used for initial data analysis. Normalization, molecular image extraction, and regions of interest were defined in SCiLS Lab MVS 2018b (Bruker Daltonics, Bremen, Germany) software typically using a mass selection window of ± 0.05 Da. AZD2858 was detected as the protonated molecular ion (M + H+) at m/z 454.2.

### 2.14. Radiation Studies

#### 2.14.1. Clonogenic Assays

Clonogenic survival assays were carried out as described previously [27]. Briefly, cells were seeded into 24-well plates at 125 cells/well in medium containing the appropriate treatment and incubated for 2 h at 37 °C before irradiation. The cells were returned to the incubator for 3 weeks. Cells were fixed with 4% PFA for 15 min and stained with methylene blue (1% *w*/*v*, 50% *v*/*v* ethanol) for 30 min. After washing in water, the colonies were counted using a Gallenkamp colony counter. Data were analyzed in Prism using the LQ model of radiation survival described by the equation S = exp(−αd−βd2), where S is the survival fraction, d is the XR dose, and α and β are constants [28].

#### 2.14.2. MTT Assay

Cells were seeded into 96-well plates (precoated with laminin for the patient-derived cell lines) and allowed to adhere for 24 h. Plates were irradiated at the doses stated, before the addition of AZD2858. Plates were subsequently incubated for 6 days at 37 °C before the addition of MTT (Sigma, Poole, UK) as per the manufacturers’ recommendations. Cells were incubated for 3 h at 37 °C and the resulting formazan crystals resuspended in DMSO. Optical density was measured at 540 nm.

### 2.15. In Vivo Models

#### 2.15.1. Subcutaneous Xenografts

BALB/c Nude mice were purchased from the breeding facility, University of Leeds. Then, 1 × 10^6^ U87-MG cells, in 50 µL PBS, were injected subcutaneously into the right flank of 7–9-week-old female mice. Once tumors were palpable (approximately 5 mm diameter), animals were randomly assigned into experimental groups. For radiation, animals were either mock-irradiated (anaesthetized but not irradiated) or received image-guided radiotherapy delivered by SARRP (Small Animal Radiation Research Platform, Xstrahl). For each treatment, a dose of 5 Gy was targeted to the tumor using a 10 × 10 mm collimator and two opposing beams on 3 consecutive days. In appropriate groups, AZD2858 10 mg/kg/day × 10 doses were also administered by oral gavage, either as single agent or in combination, starting immediately after radiation. After treatment, animal welfare was monitored daily, and tumors were measured in two planes (length and width) three times a week using calipers. Animals were euthanized once tumors attained a mean diameter of 12.5 mm. Tumor volumes were calculated using (a^2^b)/2, where a and b represent the smallest and largest dimensions of the tumor, respectively. Tumor growth rate was determined as described by Demidenko [29]. Plotting the natural log of tumor volume against time gives a straight line described by the equation lnV = α + βt, where V is tumor volume, α the y intercept (tumor starting volume), β the growth rate, and t time. Tumor doubling time (DT) was calculated using DT = ln2/β [29,30]. All experiments were performed in accordance with the Animals (Scientific Procedures) Act 1986, under license number P67C4EBE4.

#### 2.15.2. Intracranial Xenografts

Intracranial xenograft tumor assays were carried out using 6 to 8-week-old BALB/c Nude mice. Mice were stereotactically injected with 1 × 10^5^ U87 cells in a volume of 2 µL into the right striatum (2.5 mm from the midline, 2.5 mm anterior from bregma, 3 mm deep). Surgery was performed under general anesthesia using aseptic techniques. Mice were monitored daily for signs of sickness, pain, or weight loss. Six weeks after intracranial injection, animals were allocated to treatment. Irradiation was performed using a Small Animal Research Radiation Platform (SARRP; Xstrahl, UK) to give a fractionated dose of 5 Gy per day on three consecutive days (cumulative dose 15 Gy). This was achieved by firstly anaesthetizing and positioning the mouse and then performing a cone-beam CT scan. The correct segmentation was adjusted and the isocenter was aligned to the site of injection. An arc of −60 to 60 degrees with a 9 × 3 mm collimator was used to irradiate the relevant hemi-brain. Dose verification was carried out by end-to-end testing by NPL and Innovate UK. A zoomorphic mouse phantom with an intracranial cavity and tissue equivalent materials was used. Alanine pellets were inserted into the intracranial cavity and used as a reference detector. The region of interest of the pellets was calculated to obtain DVH as well as dose and volume statistics. Mice were sacrificed at the indicated endpoints and tumor and normal brain tissue was subjected to immunohistological analysis and MSI. To assess effects of treatment on dividing cell populations in normal brain, we assessed the number of dividing cells in tissue sections of sub-ventricular zone (SVZ) from control, irradiated, and drug-treated animals (1 animal, 4 sections in each condition) on day 42–53 post treatment. Sections were stained with Ki67 and positively stained cells in SVZ were counted manually, normalized per mm SVZ, and compared between conditions.

In all experiments mice were checked twice daily for adverse effects and neurological symptoms. They were weighed weekly, and animals were sacrificed if >20% weight loss occurred.

### 2.16. Statistical Analysis

In vitro data were analyzed using the Student’s *t*-test and expressed as mean ± SD or the chi-square test. For xenograft tumor analysis, the Mann–Whitney U-test was used. For Kaplan–Meier analysis, the significance was calculated using the log-rank test. *p* values of ≤0.05 were considered significant (*) and *p* values of ≤0.01 were considered highly significant (**) (***) (****). In vitro experiments were carried out in triplicate unless otherwise stated.

## 3. Results

**Figure 1:**
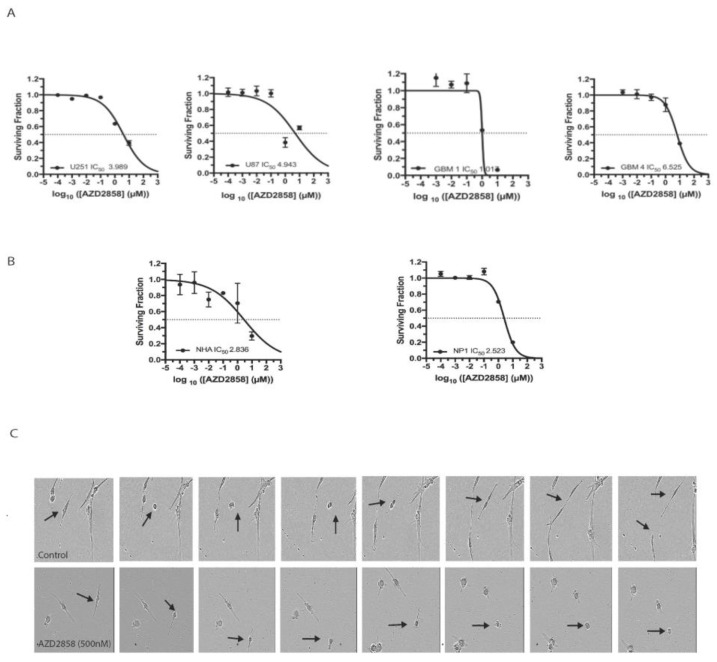
AZD2858 is cytotoxic in glioma cell lines at micromolar concentrations and induces mitotic defects at submicromolar concentrations. (**A**) The established cell lines U251 and U87 and patient-derived glioma stem cell-like cell lines GBM1 and GBM4 were treated with 10-fold dilutions of AZD2858 in triplicate. Cell viability was assessed by MTT at day 3 post treatment. (**B**) Normal brain cells including normal human astrocytes (NHA) and neural progenitor cells (NP1) were treated with 10-fold dilutions of AZD2858 in triplicate. Cell viability was assessed by MTT at day 3 post treatment. (**C**) The established cell lines U87 and U251 and the patient-derived glioma stem cell-like cell line GBM4 were treated with 1 µM or a concentration range (0.01–1 µM) of AZD2858 and imaged over 72 h or over 1 week by live cell imaging. Representative stills are shown from live cell imaging movies of the patient-derived stem cell-like cell line GBM4 mock-treated with DMSO (top panel) or treated with AZD2858 at 500 nM. This dose was chosen as no cytotoxic effects were observed on normal progenitor cells or NHA and to illustrate specific effects on mitosis in the GBM cell line. Arrows indicate mitotic cells during the duration of mitosis. Images were taken from AVI movies generated with the Incucyte imaging system. Magnification = 10×.

To assess the cytotoxic effect of GSK-3 inhibition, we treated established and patient-derived stem cell-like glioma cell lines with the specific GSK-3 inhibitor AZD2858 and assessed cell viability in MTT assays. IC_50_ doses in the range of 1.01 to 6.52 µM were established across a panel of four cell lines (two established glioblastoma cell lines, two patient-derived lines) (Figure 1A). For comparison, IC_50_ values for NP1s and NHAs grown in conditions permissive to cell division were 2.5 and 2.9 µM (Figure 1B), suggesting a similar effect on viability in dividing normal cell populations. To investigate these cytotoxic effects further, we next examined the same panel of cell lines including U251, U87, and GBM4, as well as a normal control NP1 with the Incucyte automated cell microscopy system. This assay allowed us to assess cell proliferation and to examine cell phenotype following treatment over a drug concentration range of 0.01–25 µM, including the relevant IC_50_ doses established by MTT. Detailed analysis of movies generated by live cell imaging demonstrated a pronounced effect on mitosis, with a high proportion of treated cells exhibiting multinucleation and failed mitosis at doses in low µM range through cytokinetic failure (representative images shown in Figure 1C, data in Table 1).

**Figure 2:**
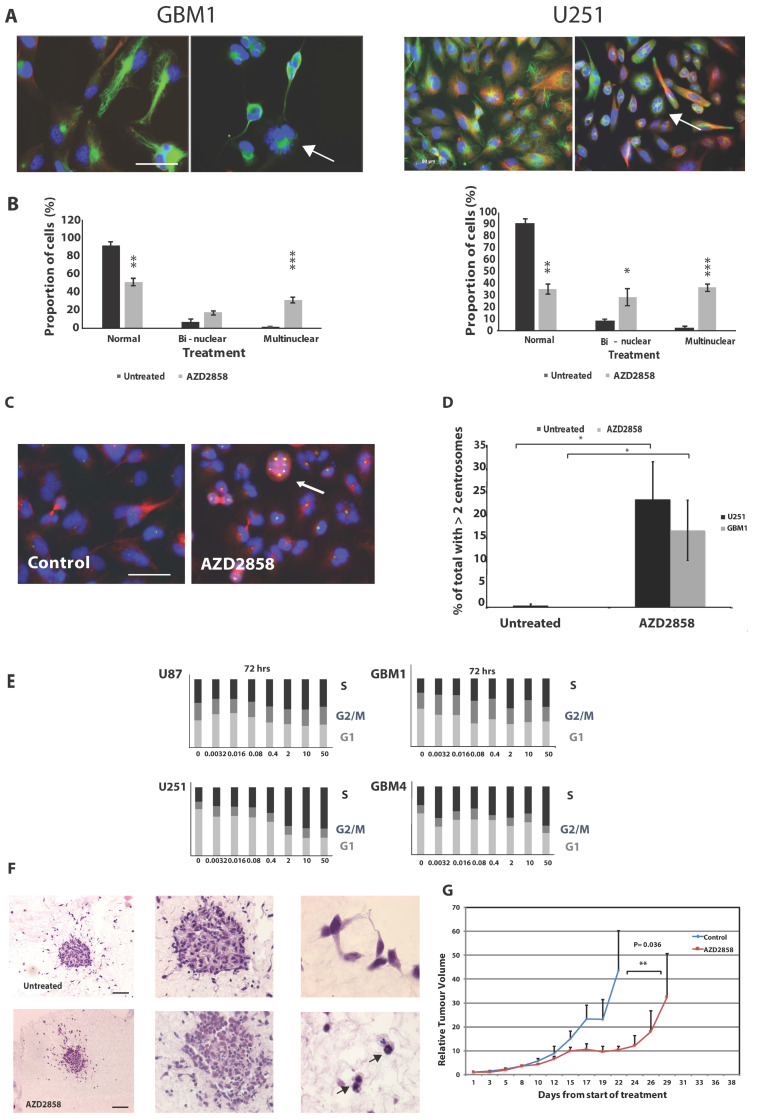
Treatment with AZD2858 induces a multinuclear phenotype in patient-derived stem cell-like lines and established glioma cell lines. (**A**) Cell lines were treated with AZD2858 at 1 µM concentration and allowed to incubate for 72 h. They were then fixed with 100% methanol and stained with anti-tubulin antibody (green) and DAPI (blue). Representative images by confocal microscopy are shown for the cell line GBM1 and U251. The arrow indicates multinucleation. Scale bar = 20 µm. (**B**) Immunofluorescence images were used for counting and quantification of cells for the presence of two (bi) or more (multi) nuclei per cell. At least 200 cells/cell line were scored. Results are shown for the patient-derived stem cell-like cell line GBM1 and the established cell line U251. (**C**) Cell lines were treated with AZD2858 at 1 µM concentration and allowed to incubate for 72 h. They were then fixed with 100% methanol and stained with anti-pericentrin antibody (green) and DAPI (blue). Representative images by confocal microscopy are shown for the cell line U251. The arrow indicates the presence of several centrosomes. Scale bar = 20 µm. (**D**) Immunofluorescence images were used for counting and quantification of cells for the presence of two or more centrosomes/cell. At least 200 cells/cell line were scored. (**E**) Established (U87, U251) and patient-derived stem cell-like cell lines (GBM1, GBM4) were mock-treated or treated with AZD2858 at 5-fold dilutions from 50 to 0.0032 µM and prepared for FACS analysis. S-phase arrest following exposure to AZD2858 was recorded after 72 h exposure in established and patient-derived stem cell-like cell lines at doses > 1 µM. (**F**) GBM4 3D spheroids embedded in collagen were treated with 500 nM AZD2858 for 72 h and then fixed with 4% paraformaldehyde for immunohistochemistry labeling. Glioma spheroids maintained within the original collagen plug were embedded in paraffin, sectioned, and then stained with H&E to visualize cellular features including the presence of bi- or multinucleated cells within the spheroids. Cells with enlarged nuclei and atypical nuclei number are indicated by the arrow (magnification left to right ×4, ×10, and ×40); scale bar = 50 µm. (**G**) In a U87 subcutaneous model, 1 × 10^6^ U87-MG cells were injected subcutaneously into the right flank of BALB/c Nude mice. Once tumors were palpable (approximately 5 mm diameter), animals were randomly assigned into experimental groups. AZD2858 was given at 10 mg/kg/day × 10 doses following fractionated irradiation (3 × 5 Gy) and tumor volumes were assessed. A significant effect on growth delay, *p* < 0.01 for AZD2858 treatment versus control was noted beyond day 17 (**). * denotes *p* ≤ 0.05, ** denotes *p* ≤ 0.01 and *** denotes *p* ≤ 0.001.

Since previous data have suggested a potential effect of GSK-3 inhibition on centrosome function, disruption of which could lead to the mitotic failure we observed, we next examined the appearance of centrosomes in treated cells by staining for the centrosome component pericentrin visualized by confocal microscopy and scored multinucleated cells in one representative patient-derived and one established cell line. These experiments revealed a marked increase in the number of cells with two or more nuclei (U251: normal—untreated vs. treated *p* = 0.0021; bi-nuclear untreated vs. treated *p* = 0.05; multinucleated untreated vs. treated *p* = 0.0007; GBM1: normal—untreated vs. treated *p* = 0.0019; bi-nucleated untreated vs. treated *p* = 0.12; multinucleated untreated versus treated *p* = 0.0001) (representative images shown in Figure 2A, data in Figure 2B) and an increase in centrosome number in treated cells compared to control, with a shift to the majority of cells exhibiting >2 centrosomes (representative images in Figure 2C, data in 2D) (U251, *p* = 0.022; GBM1, *p* = 0.034, Figure 2D). To confirm the effects on cell cycle progression, we used FACS analysis to measure cell cycle distribution post drug exposure and found a marked re-distribution of cells into S-phase after 72 h in all four cell lines, likely reflecting a brake on mitotic entry in cells with multiple centrosomes and disordered spindle formation (Figure 2E). Representative images of FACS histograms and stages of cell cycles after determination with ModFit are shown in Figure S1. Taken together, these data support the notion that GSK-3 inhibition promotes cell death in glioma cells through disruption of centrosome function and promotion of mitotic failure. To confirm that this effect was not an artefact of 2D culture, we next examined spheroids generated from glioma stem cells (GBM4). These data are shown in Figure 2F and revealed multinucleation in treated spheroids, equivalent to our observation in 2D cultures. Results indicated an increase of multinucleation from 9.05% in control spheroids to 18.33% in spheroids treated with AZD2858. We also carried out preliminary xenograft experiments using the established cell line U87 in a subcutaneous model, which demonstrated that AZD2858 given at 10 mg/kg/day × 10 doses was associated with increased growth delay compared to untreated animals (*p* < 0.05) (Figure 2G). No adverse effects on behavior or weight were observed.

Previous data have suggested that the centrosome destabilizing effect of GSK-3 beta inhibition may not be dependent on canonical signaling through Wnt-/beta-catenin or NF-κB downstream of GSK-3 [6]. To confirm the downstream signaling events following inhibition in these cell lines, we carried out Western blotting in the cell line U251, which showed the expected increase in beta-catenin and decrease in phosphorylated GSK-3 beta and - to a lesser extent - GSK-3 alpha in a dose-dependent manner (Figure S2). We also noted an increase in cyclin D gene expression levels as evidenced by real-time PCR (Figure S3A). Cyclin D is a target gene for beta-catenin promoted transcription. Based on these findings, we hypothesized that upon GSK-3 beta inhibition, pools of beta-catenin associated with the cell membrane are translocated to the nucleus where they act as transcription activators for genes with known roles in mitosis. To confirm beta-catenin translocation, we treated three cell lines (U251, GBM1, and GBM4) with AZD2858 and carried out immunofluorescence on the treated cells after incubation with the inhibitor. Beta-catenin translocation was observed within 2–72 h after treatment with AZD2858 in all cases (Figure S3B, representative data shown for U251 and GBM1).

To further probe the potential mechanism underlying these effects, we carried out a series of expression array experiments to compare changes in transcription following exposure to AZD2858. In three cell lines (U251, GBM1, GBM4), we compared expression before and at 8 and 24 h after exposure to 1 µM AZD2858. These data suggest deregulation of candidate genes with known roles in mitosis in all three cell lines (Figure S4). Genes with significant changes in expression following drug treatment across all cell lines include the protein regulator of cytokinesis 1 gene (PRC1) and the nucleolar spindle-associated protein 1 (NUSAP1), both of which have established roles in mitosis [31,32,33,34,35,36,37,38,39] (Table 2). These data suggest that a set of mitosis-associated genes are downstream targets of GSK-3 in glioma and could explain the effects we observed upon inhibition.

**Figure 3:**
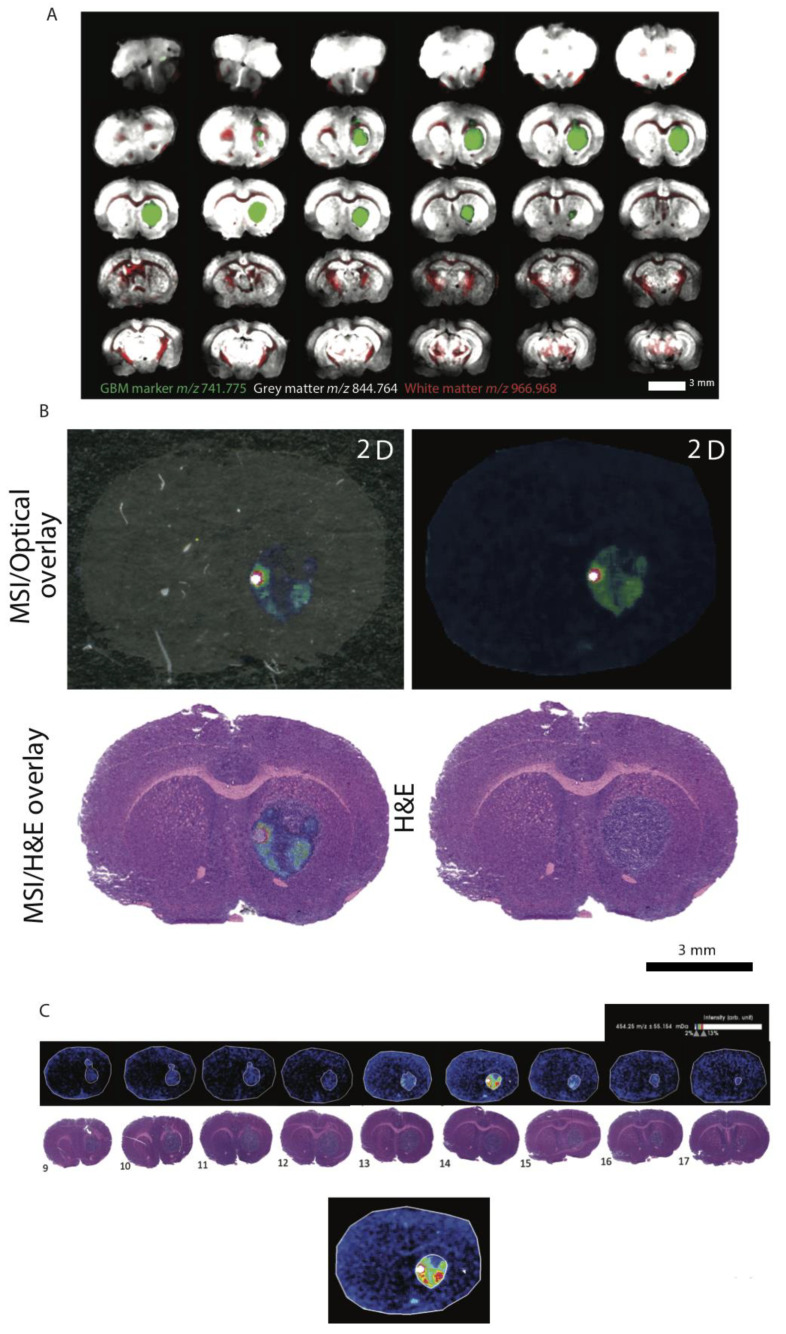
AZD2858 penetrates tumor tissue and is spatially distributed in GBM tumors in mouse brain sections. (**A**) Mouse brain tissue from BALB/c Nude mice intracranially injected with U87 cells and treated with AZD2858 were prepared for mass spectrometry imaging. Molecular markers for GBM, grey matter and white matter, were used for normal brain tissue and tumor tissue to allow enhanced visualization of tumor localization in sequential mouse brain sections. Sectioning of the full brain through 30 levels is shown. (**B**) MSI optical and H&E overlays were used to confirm AZD2858 co-localization with the tumor tissue. (**C**) Multiple tiers generated by MSI demonstrate a gradient of AZD2858 across the tumor. For the MSI/H&E overlay, image sections were selected to focus only on the GBM region in the brain (9–17 as described in (**A**) with the green marker), where AZD2858 was detected in higher abundance.

Since AZD2858 is a brain-penetrant drug that may be suitable for clinical translation, we next investigated the effect of exposure in an orthotopic xenograft model. We first confirmed agent penetration in orthotopic U87 xenografts using MALDI-MSI on whole fixed tumor-bearing brain specimens. In 3D mouse brains rendered over 30 brain sections, we identified tissue and GBM markers for molecular histology (Figure 3A). Using these markers, we could confirm that AZD2858 was detected in multiple tissue sections at low relative abundance but at 2× greater than the background (Figure 3B). AZD2858 was distinctly co-located within the tumor at abundance levels greater than for normal tissue. A concentration gradient of AZD2858 existed across the tumors (Figure 3C). Taken together, these data suggest tumor-specific uptake of AZD2858.

**Figure 4:**
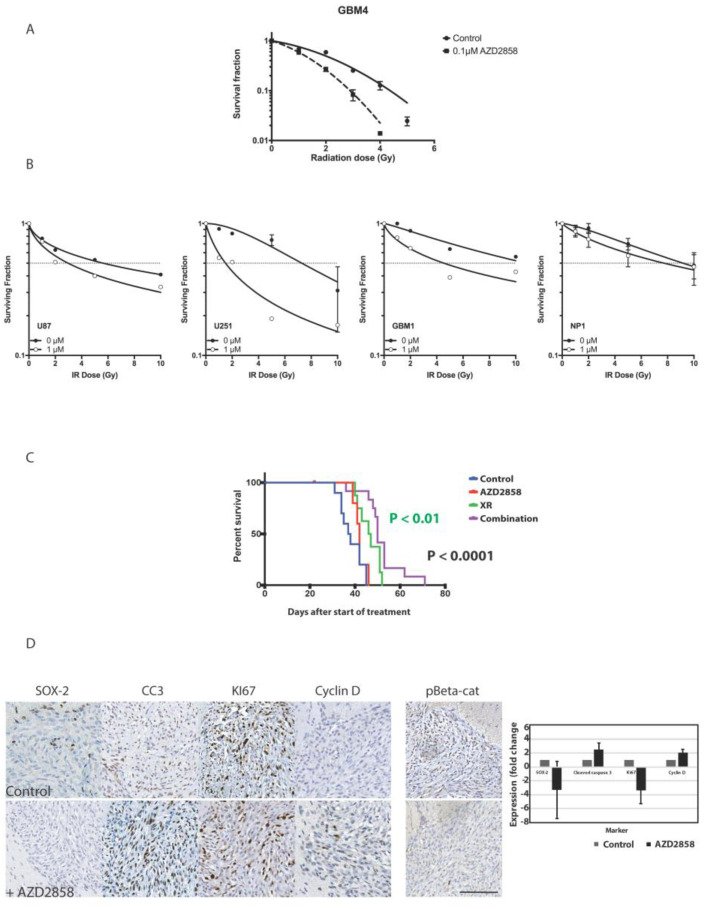
Combination treatment with AZD2858 enhances radiosensitivity in vitro and in vivo. (**A**) The patient-derived stem cell-like glioma cell line GBM4 was exposed to submicromolar concentrations of AZD2858 (0.1 µM) for 72 h after treatment with single doses of radiation (0–5 Gy). Radiosensitization was assessed in a clonogenic assay. (**B**) Additional established (U87, U251) and the patient-derived stem cell-like glioma cell line GBM1, as well as a normal cell control, neural progenitor cells (NP1), were treated with AZD2858 at 1 µM for 72 h after radiation treatment (1–10 Gy) and radiosensitization was assessed using MTT assays. (**C**) In a U87 orthotopic BALB/c Nude mice model animals were allocated to treatments six weeks after intracranial injection with either XR (5 Gy × 3), AZD2858, saline (control), or XR + AZD2858. Combination of XR with AZD2858 led to improved median survival compared to control (*p* < 0.0001) and to XR alone (*p* < 0.01). (**D**) In treated tumors from the U87 orthotopic intracranial BALB/c Nude mice model, a decrease in Sox-2 (stemness), increase in cleaved caspase 3 (apoptosis), decrease in the expression of the proliferation marker Ki67, and increase in cyclin D (cell cycle) as determined by immunohistochemistry was observed. Phosphorylated beta-catenin levels are also decreased after treatment. Representative images from untreated (control) and tumors treated with AZD2858 are shown after sectioning of harvested mouse brains and staining for the various markers by immunohistochemistry. Scale bar = 50 µm.

To establish the potential for combination with standard of care, we next assessed the effect of AZD2858 with clinically relevant radiation doses in vitro using both established and patient-derived cell lines. Since our previous data suggested that exposure to AZD2858 affected cell cycle distribution and specifically targeted dividing cells at mitosis, we reasoned that scheduling with XR may be crucial in combination experiments. Indeed, when cells were pre-exposed to AZD2858 we documented a sub-additive effect with single dose RT (Figure S5). This may be explained by cells being distributed in radioresistant cell cycle phase; for example, early S-phase, or simply due to the fact that RT and drug target overlapping cell populations, i.e., rapidly dividing cells. Consequently, in all subsequent experiments we exposed cells to AZD2858 at least 48 h following radiation. Clonogenic data using GBM4 treated with XR doses between 1 and 5 Gy and subsequently exposed to AZD2858 for 72 h at 0.1 µM concentration are shown in Figure 4A and demonstrate additive cytotoxicity. The same combination enhanced the effects of XR in U87, U251, and GBM1 cells; however, we found less effect on NP1 cells, which may be explained by higher IC_50_ and/or their slower cell cycle (Figure 4B).

**Figure 5:**
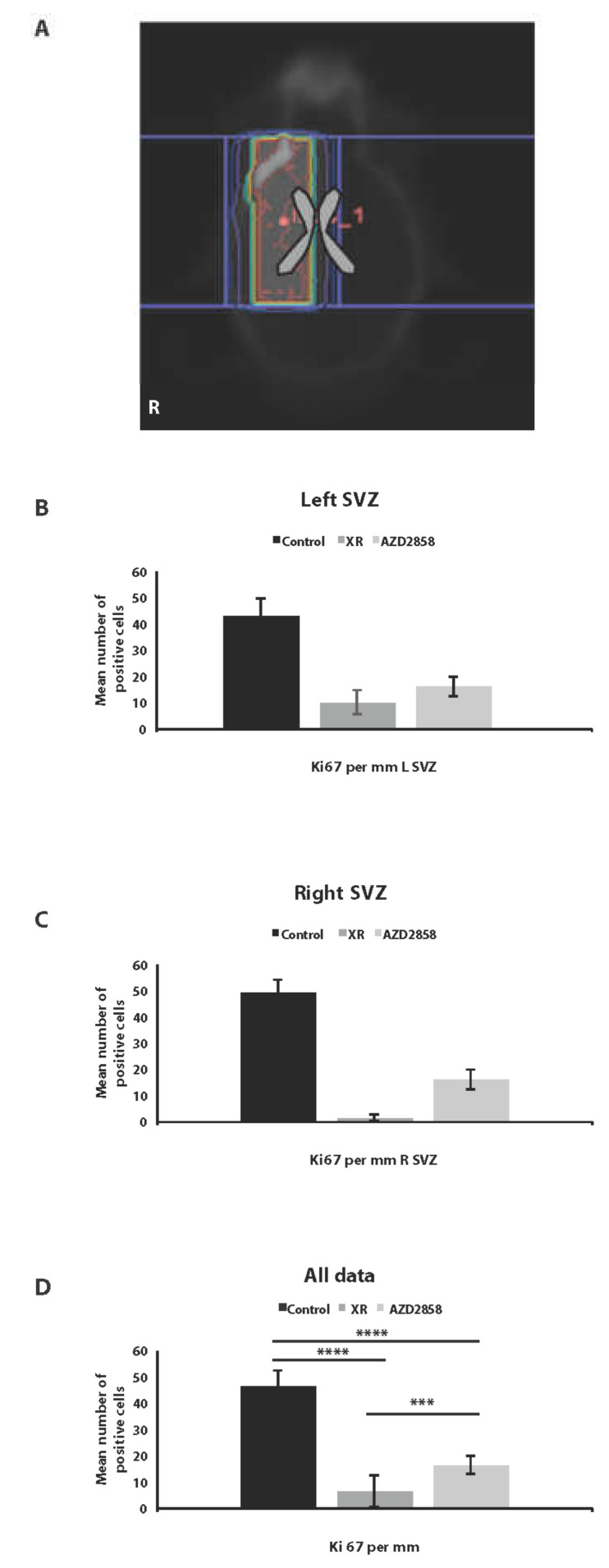
Effect of XR and AZD2858 on cell division. Ki67 staining in fixed normal brain from the U87 orthotopic intracranial BALB/c Nude mice model showing the effect of XR and AZD2858, respectively, on cell division in SVZ. Right SVZ was in the high dose RT field, left SVZ in low dose region in each case. (**A**) Representative dose contour map showing XR dose distribution in a treated animal. High dose volume is defined by yellow contour, low doses in purple. The ventricular system is outlined in grey. (**B**) Graph illustrating the result for the left SVZ, (**C**) for the right SVZ in irradiated, control, and drug-treated animals, and (**D**) all data combined (control vs. XR, *p* = 1.5 × 10^−12^; control vs. drug; *p* = 9.4 × 10^−13^; XR vs. drug, *p* = 8.2 × 10^−5^). *** denotes *p* ≤ 0.001 and **** denotes *p* ≤ 0.0001.

Having confirmed efficient delivery to intracerebral tumor site and potential sensitization to XR in vitro, we carried out additional in vivo experiments using orthotopic U87 xenografts to assess the effect of combination treatment using AZD2858 at 30 mg/kg by oral gavage following targeted 3Gy×4 days delivered using image-guided radiation (Xstrahl SARRP) compared to XR and drug alone. Combination treatment led to improved survival compared to control (*p* < 0.0001) and to XR alone (*p* < 0.01) (Figure 4C). No adverse effects on behavior or weight were observed. We also harvested brains from these mice post-mortem to assess effects of treatment on proliferation, apoptosis, and downstream targets of GSK-3 beta including beta-catenin and cyclin D in tumor tissue. A representative IHC panel is shown in Figure 4D. We confirmed by IHC analysis that there was a decrease in Ki67 expression (proliferation), increase in cleaved caspase-3 (apoptosis), decrease in SOX-2 (stemness), and increase in cyclin D indicative of a change in cyclin D turnover in treated tumors in comparison to untreated tumors and confirming our PCR results for cyclin D increase in vitro. We also noted an overall loss of phosphorylated beta-catenin, supporting that GSK-3 signaling is targeted in vivo by the activity of AZD2858. Assessment of SVZ from experimental animals in control, XR, and AZD2858 groups showed the expected effect of RT on reducing SVZ mitosis within the radiation field, and with a dose effect when right (high dose) and left (low dose) SVZ were compared. AZD2858 also reduced Ki67 positive cells in SVZ compared to control, but the effect was less marked than with XR (Figure 5A–D).

Overall, these data confirm that the phenotype we observed in glioma cell lines in which GSK-3 inhibition prevents normal mitosis through centrosome destabilization also occurs in vivo. Finally, we conclude this agent could prove an effective adjuvant to radiation since post-radiation treatment enhanced cytotoxicity in vitro and in vivo.

## 4. Discussion

High-grade gliomas remain some of the most challenging cancers to treat due to their invasive nature, chemo and radioresistance of stem cell-like subpopulations, and inevitable disease recurrence. Improved treatment relies on the discovery of novel druggable targets that promote the reversal of these characteristics and tumor cell death. GSK-3 inhibitors with CNS penetration and limited systemic toxicity are available for repurposing in glioma and we have shown in our preliminary experiments that these agents are cytotoxic but more importantly can enhance the effects of radiation in vitro and in vivo when given sequentially. Our research has highlighted that both established and patient-derived cell lines can be targeted with GSK-3 inhibitors. In vitro, we clearly demonstrated that there was a pronounced effect on mitotic events leading to cytokinetic failure and centrosomal duplication at submicromolar drug concentrations. This phenomenon has recently been reported using different GSK-3 beta inhibitors in other cancer models, supporting our data, and GSK-3 beta targeting may also explain the anti-proliferative effects of Topsentin analogues [15,40]. In addition, genes such as NUSAP1 or PRC1 featured as highly significant in our array data using AZD2858 targeting GSK-3 beta in a panel of cell lines. In gliomas, NUSAP1 is overexpressed, and expression is correlated with tumor grade and overall survival. NUSAP1 silencing results in proliferation arrest, cell death in glioma cells, and formation of supernumerary centrosomes [31,32,33,34,35,36,37,38,39]. Apart from being a substrate of several cyclin-dependent kinases (CDKs), PRC1 is essential for polarizing parallel microtubules and facilitating contractile ring assembly [32]. Even though the role of PRC1 in glioma mitosis and cytokinesis has not been investigated, data from the Human Protein Atlas indicates that there is a negative association of high expression levels of PRC1 and survival. The role of these two proteins in glioma biology, especially with regard to mitotic processes, remains to be further investigated and should be prioritized in investigating the mechanism of GSK-3 inhibition on centrosome function. Interestingly in this regard, recent reports on the effect of two GSK inhibitors, SB 415286 and RO-81220, also highlighted the role of GSK-3 in regulating mitotic checkpoint levels in multiple cancer cell lines, prolonging mitotic arrest and upstream regulating P13K/Akt signaling arcs and deacetylase sirtuin2 as a GSK-3 downstream target [41,42,43,44,45]. Our study has also highlighted the potential for combination treatment regimens. We were able to demonstrate enhanced cytotoxicity with radiation in patient-derived GBM cell lines in combination with AZD2858, with DMF in range 1.5. Predetermined agent scheduling appeared to be important, as pre-treatment with the drug followed by radiation led to radioresistance in the established cell line U251. Importantly, treatment with AZD2858 following radiation produced enhanced cytotoxicity in both established and patient-derived stem cell-like glioma cell lines with less effect in neural progenitor cells. This may be explained by slower cell cycling or a specific dependence on GSK-3 for mitotic spindle function in tumor cells, as recently suggested [46]. Our preliminary in vivo experiments (subcutaneous and orthotopic U87 models) showed proof of principle that tumor biology can be modified following one cycle of therapy. Even such limited exposure produced a modest effect of drug alone and an enhancement of the effect of fractionated radiation using growth delay and survival end-points. Our histopathology data showed significant changes in markers of proliferation, apoptosis, stemness, and cell cycle in tumors after treatment with AZD2858, supporting the cytotoxic effect of GSK-3 inhibition, which has also been recently demonstrated in other treatment-resistant tumor models including sarcoma [47,48,49,50]. Interestingly, GSK-3 inhibition may also have an important effect on anti-tumor immunity through NK cell and CD8+ T cell activation, adding further support to the notion that these agents may be effective in difficult-to-treat tumors [51,52].

## 5. Conclusions

We report for the first time the effect of the GSK-3 inhibitor AZD2858 on mitotic events in vitro and in vivo in glioma models and its enhanced activity in combination with radiation. We propose that the observed phenotype of failed cytokinesis and centrosome duplication at low drug concentrations is associated with the deregulation of mitosis-associated genes downstream of GSK-3. The specific signaling pathways that produce the observed effects on centrosome stability, cell cycle progression, and enhanced cytotoxicity require further investigation. Improved understanding of underlying signaling events will inform rational combination strategies with other agents.

## Figures and Tables

**Table 1:** AZD2858 induces cytokinetic failure and mitotic arrest in glioma cells. Treatment with AZD2858 causes prolonged mitosis, a decrease in the number of completed mitoses, and increase in cell death during mitosis. * Indicates statistically significant differences between untreated and treated cells.

Cell Line	Treatment µM AZD 2858	Average Time Spent in Mitosis (Mins)	% Total Cells Completed Mitosis	% Total Cells with Incomplete Mitosis
U87	Control	151	100%	0%
	+1 µM	424 *	16%	84% *
U251	Control	265	100%	0%
	+1 µM	795	66%	34% *
NP1	Control	182	100%	0%
	+0.01 µM	268 *	83%	17%
	+0.1 µM	200	100%	0%
	+1 µM	182	80%	20%
GBM4	Control	145	100%	0%
	+0.01 µM	133	86%	14%
	+0.1 µM	203	100%	0%
	+1 µM	150	0%	100% *

**Table 2:** Treatment with 1 µM AZD2858 leads to the deregulation of genes associated with mitosis in the patient-derived cell line GBM1 as determined in an expression microarray (top hits are shown in the table). The highlighted genes in grey are genes with known functions in mitosis.

Average Expression	LOGFC	*p*-Value	FDR	Probe	Systematic Name	Symbol	Entrez
**14.4**	−0.39	1.47 × 10^−7^	6.58 × 10^−6^	A_33_P3317523	NM_203401	STMN1	3925
**14.0**	0.40	7.76 × 10^−11^	1.49 × 10^−8^	A_24_P322444	NM_153719	NUP62	23636
**13.2**	−0.26	1.50 × 10^−5^	0.00026	A_23_P206059	NM_003981	PRC1	9055
**12.8**	−0.50	2.10 × 10^−8^	1.42 × 10^−6^	A_23_P406424	NM_175744	RHOC	389
**12.1**	−0.60	8.53 × 10^−11^	1.60 × 10^−8^	A_33_P3350488	NM_016359	NUSAP1	51203
**12.1**	−0.27	2.21 × 10^−5^	0.00036	A_23_P48835	NM_138555	KIF23	9493
**11.6**	0.24	6.76 × 10^−5^	0.00091	A_23_P100868	NM_001033580	MYO19	80179
**11.6**	−0.41	1.47 × 10^−6^	4.08 × 10^−5^	A_23_P420551	NM_007174	CIT	11113
**11.1**	−0.33	2.28 × 10^−6^	5.78 × 10^−5^	A_33_P3298356	NM_001206651	SH3GLB1	51100
**11.1**	−0.34	5.33 × 10^−5^	0.00075	A_23_P115872	NM_018131	CEP55	55165
**11.0**	−0.39	1.12 × 10^−7^	5.26 × 10^−6^	A_23_P89410	NM_003766	BECN1	8678
**10.9**	−0.36	5.94 × 10^−5^	0.00082	A_23_P115872	NM_018131	CEP55	55165
**10.5**	−0.25	5.71 × 10^−5^	0.00080	A_23_P65930	NM_001077268	ZFYVE19	84936
**10.1**	−0.55	1.07 × 10^−6^	3.17 × 10^−5^	A_23_P35399	NM_145869	ANXA11	311
**10.0**	−0.88	6.59 × 10^−7^	2.16 × 10^−5^	A_23_P18939	NM_002890	RASA1	5921
**9.6**	0.26	2.99 × 10^−6^	7.19 × 10^−5^	A_33_P3421748	ENST00000381679	SON	6651
**9.1**	0.60	2.85 × 10^−5^	0.00044	A_33_P3288110	NM_001005476	PKP4	8502
**8.8**	−0.44	4.34 × 10^−5^	0.00063	A_33_P3257140	NM_004850	ROCK2	9475
**8.7**	0.52	6.76 × 10^−6^	0.00014	A_24_P286935	NM_004311	ARL3	403
**7.8**	0.42	2.79 × 10^−6^	6.79 × 10^−5^	A_24_P173124	NM_144997	FLCN	201163
**7.1**	−0.27	5.76 × 10^−5^	0.00080	A_23_P254404	NM_153271	SNX33	257364

## Data Availability

The data presented in this study are available on request from the corresponding authors.

## Supplementary Materials

The following are available online at https://www.mdpi.com/2072-6694/13/23/5939/s1, Figure S1: Histogram plots obtained for the established cell line U87 after determination of cell cycle stages with ModFit, Figure S2: Western blots of protein lysates of the established cell line U251 treated with AZD2858, Figure S3: AZD2858 promotes an increase in cyclin D and leads to beta-catenin nuclear translocation, Figure S4: Microarray data analysis reveals the deregulation of mitosis-associated genes after treatment with AZD2858, Figure S5: The effect of pre-exposure to AZD2858 on sensitivity to radiation treatment.

## References

[1] Bao S., Wu Q., McLendon R.E., Hao Y., Shi Q., Hjelmeland A.B., Dewhirst M.W., Bigner D.D., Rich J.N. (2006). Glioma stem cells promote radioresistance by preferential activation of the DNA damage response. Nature.

[2] Carruthers R.D., Ahmed S.U., Ramachandran S., Strathdee K., Kurian K.M., Hedley A., Gomez-Roman N., Kalna G., Neilson M., Gilmour L. (2018). Replication Stress Drives Constitutive Activation of the DNA Damage Response and Radioresistance in Glioblastoma Stem-like Cells. Cancer Res..

[3] Ong D., Hu B., Ho Y.W., Sauvé C.G., Bristow C.A., Wang Q., Multani A.S., Chen P., Nezi L., Jiang S. (2017). PAF promotes stemness and radioresistance of glioma stem cells. Proc. Natl. Acad. Sci. USA.

[4] Schulz A., Meyer F., Dubrovska A., Borgmann K. (2019). Cancer Stem Cells and Radioresistance: DNA Repair and Beyond. Cancers.

[5] Liang M.H., Chuang D.M. (2016). Differential roles of glycogen synthase kinase-3 isoforms in the regulation of transcriptional activation. JBC.

[6] Wagner F.F., Benajiba L., Campbell A.J., Weïwer M., Sacher J.R., Gale J.P., Ross L., Puissant A., Alexe G., Conway A. (2018). Exploiting an Asp-Glu “switch” in glycogen synthase kinase 3 to design paralog-selective inhibitors for use in acute myeloid leukemia. Sci. Trans. Med..

[7] Ugolkov A., Qiang W., Bondarenko G., Procissi D., Gaisina I., James C.D., Chandler J., Kozikowski A., Gunosewoyo H., O’Halloran T. (2017). Combination Treatment with the GSK-3 Inhibitor 9-ING-41 and CCNU Cures Orthotopic Chemoresistant Glioblastoma in Patient-Derived Xenograft Models. Trans. Oncol..

[8] Miyashita K., Kawakami K., Nakada M., Mai W., Shakoori A., Fujisawa H., Hayashi Y., Hamada J., Minamoto T. (2009). Potential therapeutic effect of glycogen synthase kinase 3beta inhibition against human glioblastoma. Clin. Cancer Res..

[9] Pecoraro C., Faggion B., Balboni B., Carbone D., Peters G.J., Diana P., Assaraf Y.G., Giovannetti E. (2021). GSK3beta as a novel promising target to overcome chemoresistance in pancreatic cancer. Drug Resist. Update.

[10] Nakada M., Minamoto T., Pyko I.V., Hayashi Y., Hamada J.-I. (2011). The Pivotal Roles of GSK-3β in Glioma Biology.

[11] Abdullah L.N., Chow E.K.H. (2013). WNT signalling pathway and chemoresistance: Mechanisms of chemoresistance in cancer stem cells. Clin. Transl. Med..

[12] Tan Z., Song L., Wu W., Zhou Y., Zhu J., Wu G., Cao L., Song J., Li J., Zhang W. (2018). TRIM14 promotes chemoresistance in gliomas by activating Wnt/β-catenin signaling via stabilizing Dvl2. Oncogene.

[13] Li H., Li J., Zhang G., Da Q., Chen L., Yu S., Zhou Q., Weng Z., Xin Z., Shi L. (2019). HMGB1-induced p62 overexpression promotes Snail-mediated epithelial-mesenchymal transition in Glioblastoma cells via the degradation of GSK-3beta. Theranostics.

[14] Atkins R.J., Stylli S.S., Luwor R.B., Kaye A.H., Hovens C.M. (2013). Glycogen synthase kinase-3β (GSK-3β) and its dysregulation in glioblastoma multiforme. J. Clin. Neurosci..

[15] Yoshino Y., Ishioka C. (2015). Inhibition of glycogen synthase kinase-3 beta induces apoptosis and mitotic catastrophe by disrupting centrosome regulation in cancer cells. Sci. Rep..

[16] Wu X., Stenson M., Abeykoon J., Nowakowski K., Zhang L., Lawson J., Wellik L., Li Y., Krull J., Wenzl K. (2019). Targeting glycogen synthase kinase 3 for therapeutic benefit in lymphoma. Blood.

[17] Wurdak H., Zhu S., Romero A., Lorger M., Watson J., Chiang C.Y., Zhang J., Natu V.S., Lairson L.L., Walker J.R. (2010). An RNAi screen identifies TRRAP as a regulator of brain tumor-initiating cell differentiation. Cell Stem Cell.

[18] Polson E.S., Kuchler V.B., Abbosh C., Ross E.M., Mathew R.K., Beard H.A., da Silva B., Holding A.N., Ballereau S., Chuntharpursat-Bon E. (2018). KHS101 disrupts energy metabolism in human glioblastoma cells and reduces tumor growth in mice. Sci. Trans. Med..

[19] Cheng V., Esteves F., Chakrabarty A., Cockle J., Short S., Brüning-Richardson A. (2015). High-content analysis of tumour cell invasion in three-dimensional spheroid assays. Oncoscience.

[20] Gilmour P.S., O’Shea P.J., Fagura M., Pilling J.E., Sanganee H., Wada H., Courtney P.F., Kavanagh S., Hall P.A., Escott K.J. (2013). Human stem cell osteoblastogenesis mediated by novel glycogen synthase kinase 3 inhibitors induces bone formation and a unique bone turnover biomarker profile in rats. Toxicol. Appl. Pharmacol..

[21] Cockle J.V., Picton S., Levesley J., Ilett E., Carcaboso A.M., Short S., Steel L.P., Melcher A., Lawler S.E., Brüning-Richardson A. (2015). Cell migration in paediatric glioma; characterisation and potential therapeutic targeting. Br. J. Cancer.

[22] Levesley J., Steele L., Brüning-Richardson A., Davison A., Zhou J., Ding C., Lawler S., Short S.C. (2018). Selective BCL-XL inhibition promotes apoptosis in combination with MLN8237 in medulloblastoma and pediatric glioblastoma cells. Neuro-Oncology.

[23] Ritchie M.E., Phipson B., Wu D., Hu Y., Law C.W., Shi W., Smyth G.K. (2015). limma powers differential expression analyses for RNA-sequencing and microarray studies. Nucleic Acids Res..

[24] Silver J.D., Ritchie M.E., Smyth G.K. (2009). Microarray background correction: Maximum likelihood estimation for the normal-exponential convolution. Biostatistics.

[25] Smyth G.K., Speed T. (2003). Normalization of cDNA microarray data. Methods.

[26] Yang Y.H., Thorne N.P., Goldstein D.R. (2003). Normalization for two-color cDNA microarray data. Science and Statistics: A Festschrift for Terry Speed, IMS Lecture Notes—Monograph Series.

[27] Franken N.A., Rodermond H.M., Stap J., Haveman J., van Bree C. (2006). Clonogenic assay of cells in vitro. Nat. Protoc..

[28] Douglas B.G., Fowler J.F. (1976). The effect of multiple small doses of X-rays on skin reactions in the mouse and a basic interpretation. Radiat. Res..

[29] Demidenko E. (2010). Three endpoints of in vivo tumour radiobiology and their statistical estimation. Int. J. Radiat. Biol..

[30] Mehrara E., Forssell-Aronsson E., Ahlman H., Bernhardt P. (2007). Specific growth rate versus doubling time for quantitative characterization of tumor growth rate. Cancer. Res..

[31] EntrezGene: PRC1proteinregulatorofcytokinesis1. https://www.genenames.org/data/gene-symbolreport/#!/hgnc_id/HGNC:9341.

[32] Jiang W., Jimenez G., Wells N.J., Hope T.J., Wahl G.M., Hunter T., Fukunaga R. (1998). PRC1: A human mitotic spindle-associated CDK substrate protein required for cytokinesis. Mol. Cell..

[33] EntrezGene: NUSAP1. https://www.ncbi.nlm.nih.gov/gene/51203.

[34] Zhu T., Xie P., Gao Y.F., Huang M.S., Li X., Zhang W., Zhou H.H., Liu Z.Q. (2018). Nucleolar and spindle-associated protein 1 is a tumor grade correlated prognosis marker for glioma patients. CNS Neurosci. Ther..

[35] Wu X., Xu B., Yang C., Wang W., Zhong D., Zhao Z., He L., Hu Y., Jiang L., Li J. (2017). Nucleolar and spindle associated protein 1 promotes the aggressiveness of astrocytoma by activating the Hedgehog signaling pathway. J. Exp. Clin. Cancer Res..

[36] Barr F.A., Gruneberg U. (2007). Cytokinesis: Placing and making the final cut. Cell.

[37] Shimo A., Nishidate T., Ohta T., Fukuda M., Nakamura Y., Katagiri T. (2007). Elevated expression of protein regulator of cytokinesis 1, involved in the growth of breast cancer cells. Cancer Sci..

[38] Iyer J., Moghe S., Furukawa M., Tsai M.Y. (2011). What’s Nu(SAP) in mitosis and cancer?. Cell Signal..

[39] Mills C.A., Suzuki A., Arceci A., Mo J.Y., Duncan A., Salmon E.D., Emanuele M.J. (2017). Nucleolar and spindle-associated protein 1 (NUSAP1) interacts with a SUMO E3 ligase complex during chromosome segregation. J. Biol. Chem..

[40] Carbone D., Parrino B., Cascioferro S., Pecoraro C., Giovannetti E., Di Sarno V., Musella S., Auriemma G., Cirrincione G., Diana P. (2021). 1,2,4-Oxadiazole Topsentin analogs with antiproliferative activity against pancreatic cancer cells, targeting GSK3beta kinase. Chem. Med. Chem..

[41] Yang E.S., Nowsheen S., Wang T., Thotala D.K., Xia F. (2011). Glycogen synthase kinase 3beta inhibition enhances repair of DNA double-strand breaks in irradiated hippocampal neurons. Neuro-Oncology.

[42] Shimura T., Noma N., Oikawa T., Ochiai Y., Kakuda S., Kuwahara Y., Takai Y., Takahashi A., Fukumoto M. (2012). Activation of the AKT/cyclin D1/Cdk4 survival signaling pathway in radioresistant cancer stem cells. Oncogenesis.

[43] Minniti G., Muni R., Lanzetta G., Marchetti P., Enrici R.M. (2009). Chemotherapy for glioblastoma: Current treatment and future perspectives for cytotoxic and targeted agents. Anticancer Res..

[44] Kotliarova S., Pastorino S., Kovell L.C., Kotliarov Y., Song H., Zhang W., Bailey R., Maric D., Zenklusen J.C., Lee J. (2008). Glycogen synthase kinase-3 inhibition induces glioma cell death through c-MYC, nuclear factor-kappaB, and glucose regulation. Cancer Res..

[45] Atkins R.J., Dimou J., Paradiso L., Morokoff A.P., Kaye A.H., Drummond K.J., Hovens C.M. (2012). Regulation of glycogen synthase kinase-3 beta (GSK-3β) by the Akt pathway in gliomas. J. Clin. Neurosci..

[46] Rashid M.S., Mazur T., Ji W., Liu S.T., Taylor W.R. (2018). Analysis of the role of GSK3 in the mitotic checkpoint. Sci. Rep..

[47] Williams S.P., Nowicki M.O., Liu F., Press R., Godlewski J., Abdel-Rasoul M., Kaur B., Fernandez S.A., Chiocca E.A., Lawler S.E. (2011). Indirubins decrease glioma invasion by blocking migratory phenotypes in both the tumor and stromal endothelial cell compartments. Cancer Res..

[48] Kanehira M., Katagiri T., Shimo A., Takata R., Shuin T., Miki T., Fujioka T., Nakamura Y. (2007). Oncogenic role of MPHOSPH1, a cancer-testis antigen specific to human bladder cancer. Cancer Res..

[49] Dong Z. (2018). Nuclear GSK3β induces DNA double-strand break repair by phosphorylating 53BP1 in glioblastoma. Int. J. Oncol..

[50] Abe K., Yamamoto N., Domoto T., Bolidong D., Hayashi K., Takeuchi A., Miwa S., Igarashi K., Inatani H., Aoki Y. (2020). Glycogen synthase kinase 3β as a potential therapeutic target in synovial sarcoma and fibrosarcoma. Cancer Sci..

[51] Augello G., Emma M.R., Cusimano A., Azzolina A., Montalto G., McCubrey J.A., Cervello M. (2020). The Role of GSK-3 in Cancer Immunotherapy: GSK-3 Inhibitors as a new frontier in cancer treatment. Cells.

[52] Taylor A., Rudd C.E. (2020). Glycogen synthase kinase 3 (GSK-3) controls T-cell motility and interactions with antigen presenting cells. BMC Res. Notes.

