# Correction: Brüning-Richardson et al. GSK-3 Inhibition Is Cytotoxic in Glioma Stem Cells through Centrosome Destabilization and Enhances the Effect of Radiotherapy in Orthotopic Models. *Cancers* 2021, *13*, 5939

**DOI:** 10.3390/cancers14153789

**Published:** 2022-08-04

**Authors:** Anke Brüning-Richardson, Gary C. Shaw, Daniel Tams, Tim Brend, Hitesh Sanganee, Simon T. Barry, Gregory Hamm, Richard J. A. Goodwin, John G. Swales, Henry King, Lynette Steele, Ruth Morton, Anastasia Widyadari, Thomas A. Ward, Filomena Esteves, Marjorie Boissinot, Georgia Mavria, Alastair Droop, Sean E. Lawler, Susan C. Short

**Affiliations:** 1 Leeds Institute of Medical Research at St James’s, University of Leeds, Leeds LS9 7TF, UK; 2 Discovery Sciences BioPharmaceuticals R&D, AstraZeneca, Cambridge CB2 8PA, UK; 3 Bioscience, Early Oncology, Oncology R&D, AstraZeneca, Cambridge CB2 8PA, UK; 4 Imaging and Data Analytics, Clinical Pharmacology and Safety Sciences, BioPharmaceuticals R&D, AstraZeneca, Cambridge CB2 8PA, UK; 5 Leeds MRC Medical Bioinformatics Centre, University of Leeds, Leeds LS9 7TF, UK; 6 Pathology & Laboratory Medicine, Brown University Cancer Center, Brown University, Providence, RI 02903, USA

The authors wish to make the following corrections to this paper [1]:

**1.**  **Addition of an Author**

*Dr. Georgia Mavria was not included as an author in the original publication, the correct authorship is as below:*

Anke Brüning-Richardson ^1^^,^*, Gary C. Shaw ^1^, Daniel Tams ^1^, Tim Brend ^1^, Hitesh Sanganee ^2^, Simon T. Barry ^3^, Gregory Hamm ^4^, Richard J. A. Goodwin ^4^, John G. Swales ^4^, Henry King ^1^, Lynette Steele ^1^, Ruth Morton ^1^, Anastasia Widyadari ^1^, Thomas A. Ward ^1^, Filomena Esteves ^1^, Marjorie Boissinot ^1^, Georgia Mavria ^1^, Alastair Droop ^5^, Sean E. Lawler ^6^ and Susan C. Short ^1,^*

*The author contribution should also be updated to include GM as below:*

Conceptualization, S.C.S. and A.B.-R.; methodology, A.B.-R., G.C.S., D.T., T.B., H.S., S.T.B., G.H., R.J.A.G., J.G.S., H.K., L.S., R.M., A.W., T.A.W., F.E., M.B., A.D., G.M.; software, A.D.; validation, S.C.S., S.E.L.; formal analysis, S.C.S., G.C.S., A.B.-R.; investigation, A.B.-R., G.C.S., D.T., T.B., H.S., S.T.B., G.H., R.J.A.G., J.G.S., H.K., L.S., R.M., A.W., T.A.W., F.E., M.B., A.D.; resources, H.S., S.T.B., G.H., R.J.A.G.; data curation, S.C.S., A.D.; writing—original draft preparation, A.B.-R., S.C.S., S.E.L.; writing—review and editing, S.C.S., S.E.L.; visualization, A.B.-R., S.C.S.; supervision, S.C.S.; project administration, S.C.S.; funding acquisition, S.C.S., A.B.-R. All authors have read and agreed to the published version of the manuscript.

**2.**  **Email Correction**

The correct email address of Susan C. Short is s.c.short@leeds.ac.uk

**3.**  **Text Correction**

We have also been alerted to an error in affiliation in the text of the paper: In Section 2.1 Cell lines, sentence 2 and 3: In addition, patient-derived cell lines GBM1 and GBM4 [15] were included in this study. Neural progenitor cells (NP1) and normal human astrocytes (NHA) were obtained from Dr Heiko Wurdak (Leeds Institute of Cancer and Pathology). It should be replaced with the following: The patient-derived tumour models GBM1, GBM4, and the adult human brain progenitor line NP1 were a kind gift from H. Wurdak (Stem Cell and Brain Tumour Group, University of Leeds, Leeds, UK) and were used as previously described in [17,18].

The authors apologize for any inconvenience caused and state that the scientific conclusions are unaffected. The original article has been updated.
