# Supplementary material for: GSK-3 Inhibition Is Cytotoxic in Glioma Stem Cells through Centrosome Destabilization and Enhances the Effect of Radiotherapy in Orthotopic Models
Source: Cancers (Basel). 2021 Nov 25;13(23):5939. doi: 10.3390/cancers13235939 (PMC8657225; doi:10.3390/cancers13235939)
Supplement: Supplementary file 1 [file cancers-13-05939-s001.zip › Supplemental figures and legends/Figure legends for supplemental figures 241121 AZ paper.pdf]

Figure S1. Histogram plots obtained for the established cell line U87 after determination of cell cycle stages with ModFit. The data obtained for the control (no treatment, (**A**)) indicates that U87 display little polyploidy, and this represents the accurate control. (**B**) When treated with AZD2858 (1 μM), total aneuploidy does not change in response to exposure to the inhibitor.

Figure S2. (A) Western blots of protein lysates of the established cell line U251 treated with AZD2858 at varying concentrations (0, 0.1, 1, and 10 μM) over 72 h were analyzed for protein levels for beta-catenin, GSK-3 alpha, GSK-3 beta, phosphorylated GSK-3 alpha, phosphorylated GSK-3 beta, Akt, or phosphorylated Akt over 72 h. TACC3 is a centrosomal marker. Beta-actin was used as internal loading control. (**B)** Whole Western profiles for b-catenin, GSK-alpha (GSK3α), GSK-beta (GSK3β), and TACC3. **(C)** Whole Western profiles for p-GSK3-alpha (p-GSK3α), and p-GSK-3 beta (p-GSK3β). **(D)** Whole Western profiles for Akt and p-Akt**. (E)** Whole Western profiles for beta actin control. MW values indicate predicted molecular weights.

Figure S3. AZD2858 promotes an increase in cyclin D and leads to beta-catenin nuclear translocation in established and patient-derived stem cell-like glioma cell lines. (**A**)The established glioma cell line U251 was treated with 10-fold concentrations of AZD2858 (0.01–10 μM) for 72 h. Cyclin D expression was assessed by real-time PCR. (**B**) The established cell line U251 and patient-derived stem cell-like glioma cell lines GBM1 and GBM4 were treated for 2 h with AZD2858 at 500 nM and then fixed with 4% paraformaldehyde for immunofluorescence labeling. Arrow indicates beta-catenin cellular localization (green—beta-catenin antibody; red—actin phalloidin label; blue—DAPI; ×63, scale bar = 10 μm).

Figure S4. Microarray data analysis reveals the deregulation of mitosis-associated genes after treatment of established and patient-derived stem cell-like glioma cell lines with AZD2858. Cells from three different cell lines (U251, GBM1, and GBM4) were treated with 1 μM AZD2858 or mock-treated with 0.01% DMSO in fresh media. At 8 and 24 h intervals, cells were harvested and snap frozen on dry ice (in triplicate) for microarray analysis. Highly significant gene hits were collated in a Venn diagram, with green indicating mitotic genes that were upregulated in response to the treatment and red that were downregulated at 8 and 24 h after treatment. A yellow result indicates a different result (either up or downregulated) depending on the cell line.

Figure S5. The effect of pre-exposure to AZD2858 on sensitivity to radiation treatment. U251 cells were pre-exposed to AZD2858 for 72 h and then treated with single dose radiation (1–5Gy). Survival was measured using a clonogenic assay.
