# Supplementary figures and images for: GSK-3 Inhibition Is Cytotoxic in Glioma Stem Cells through Centrosome Destabilization and Enhances the Effect of Radiotherapy in Orthotopic Models
Source: Cancers (Basel). 2021 Nov 25;13(23):5939. doi: 10.3390/cancers13235939 (PMC8657225; doi:10.3390/cancers13235939)

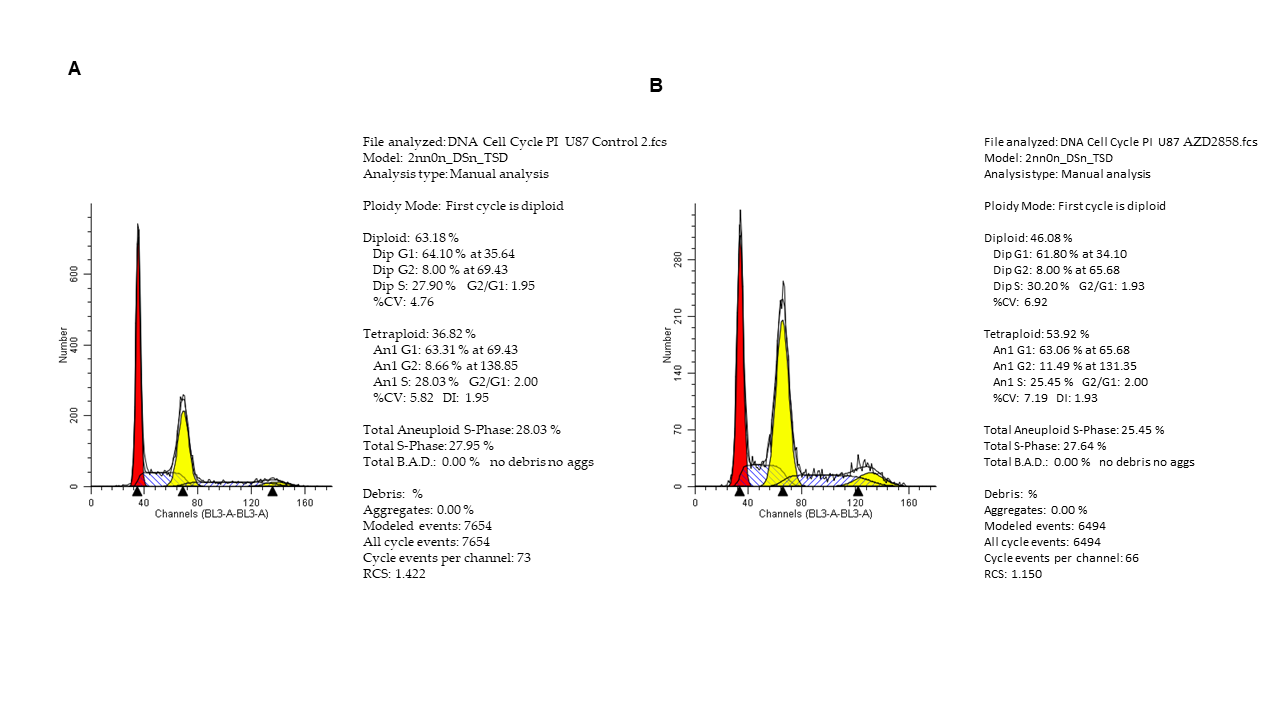

Supplement: Supplementary file 1 [file cancers-13-05939-s001.zip › Supplemental figures and legends/Supplemental figure 1 241121.png]

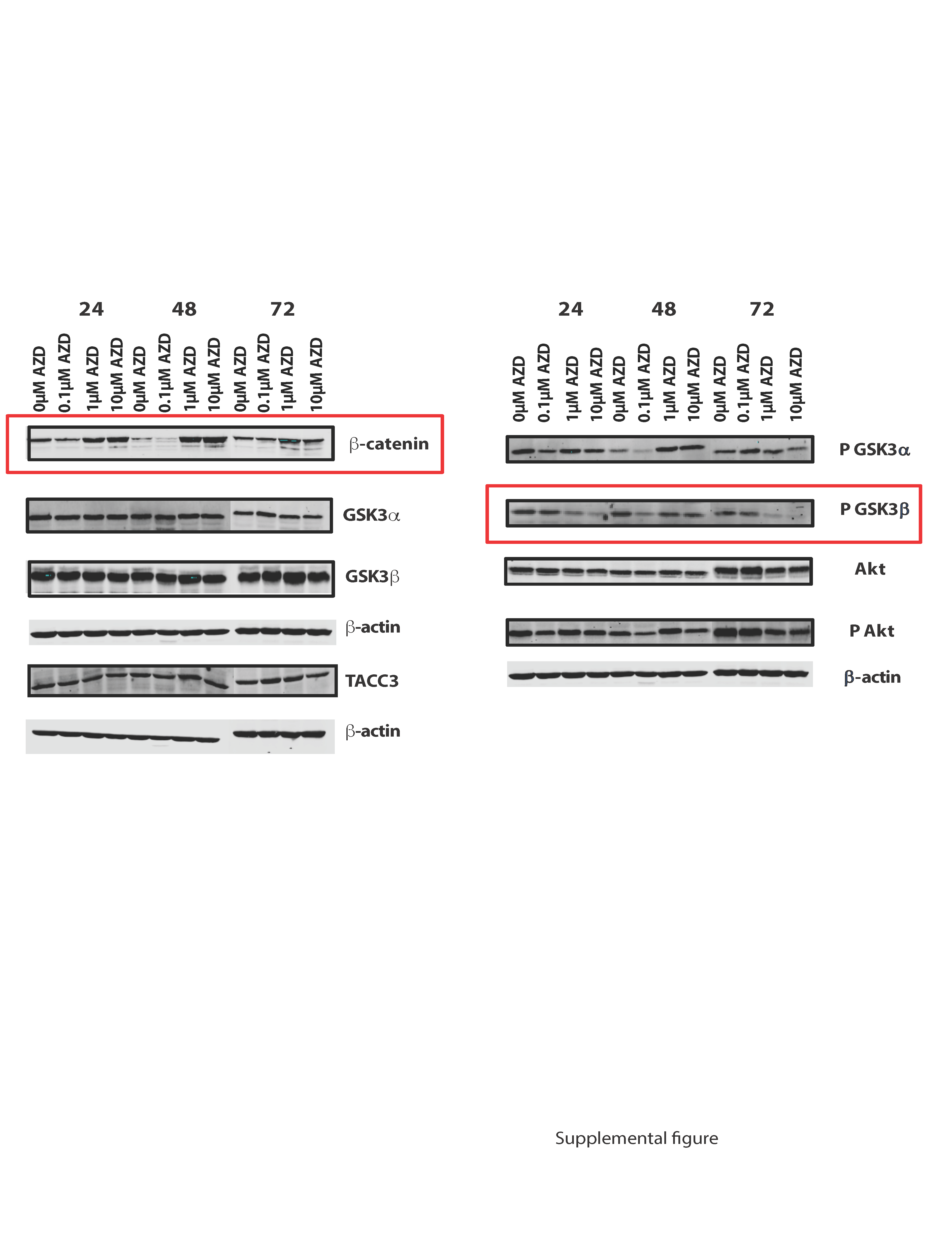

Supplement: Supplementary file 1 [file cancers-13-05939-s001.zip › Supplemental figures and legends/Supplemental figure 2A final 251121.png]

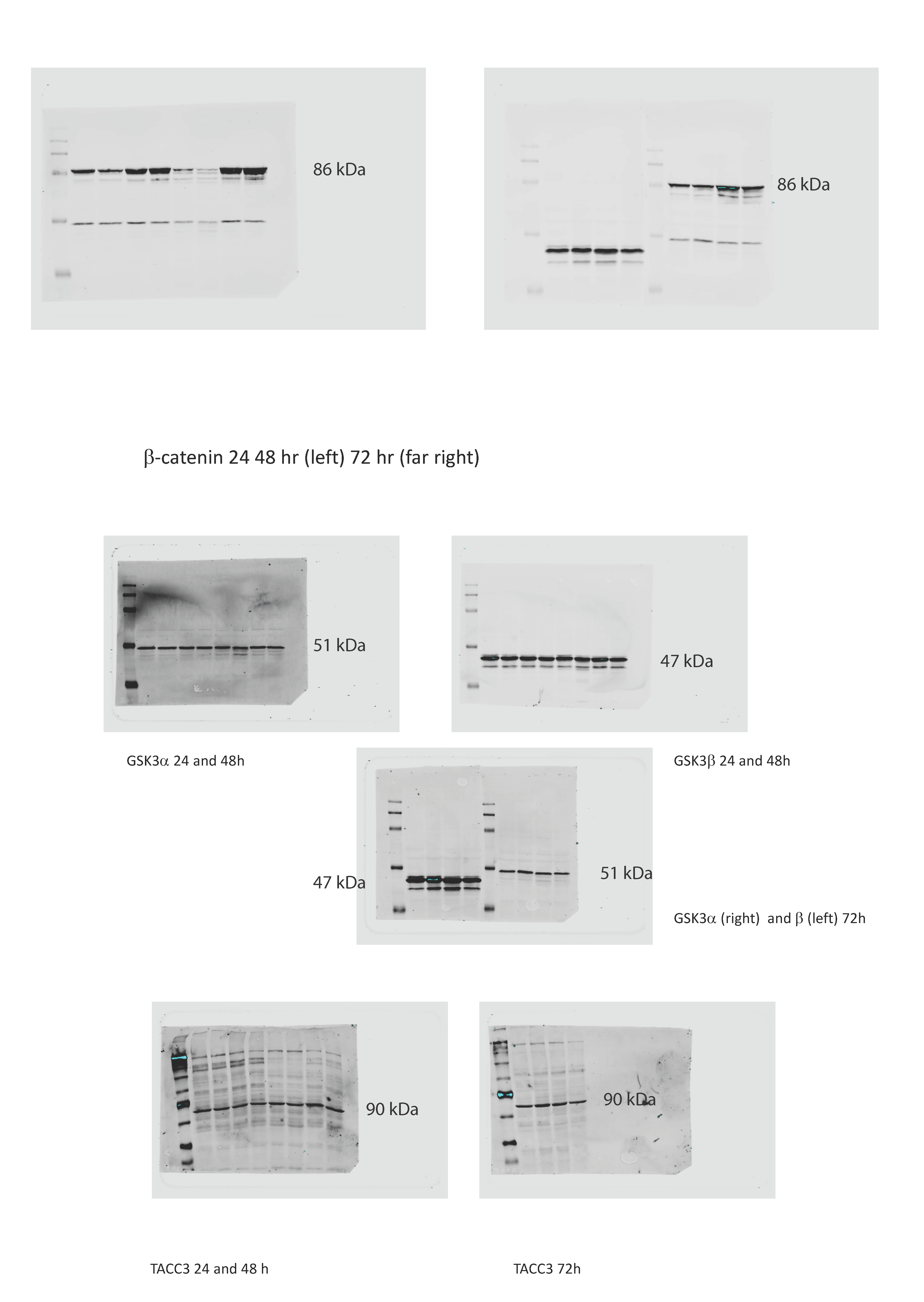

Supplement: Supplementary file 1 [file cancers-13-05939-s001.zip › Supplemental figures and legends/Supplemental figure 2B 241121.png]

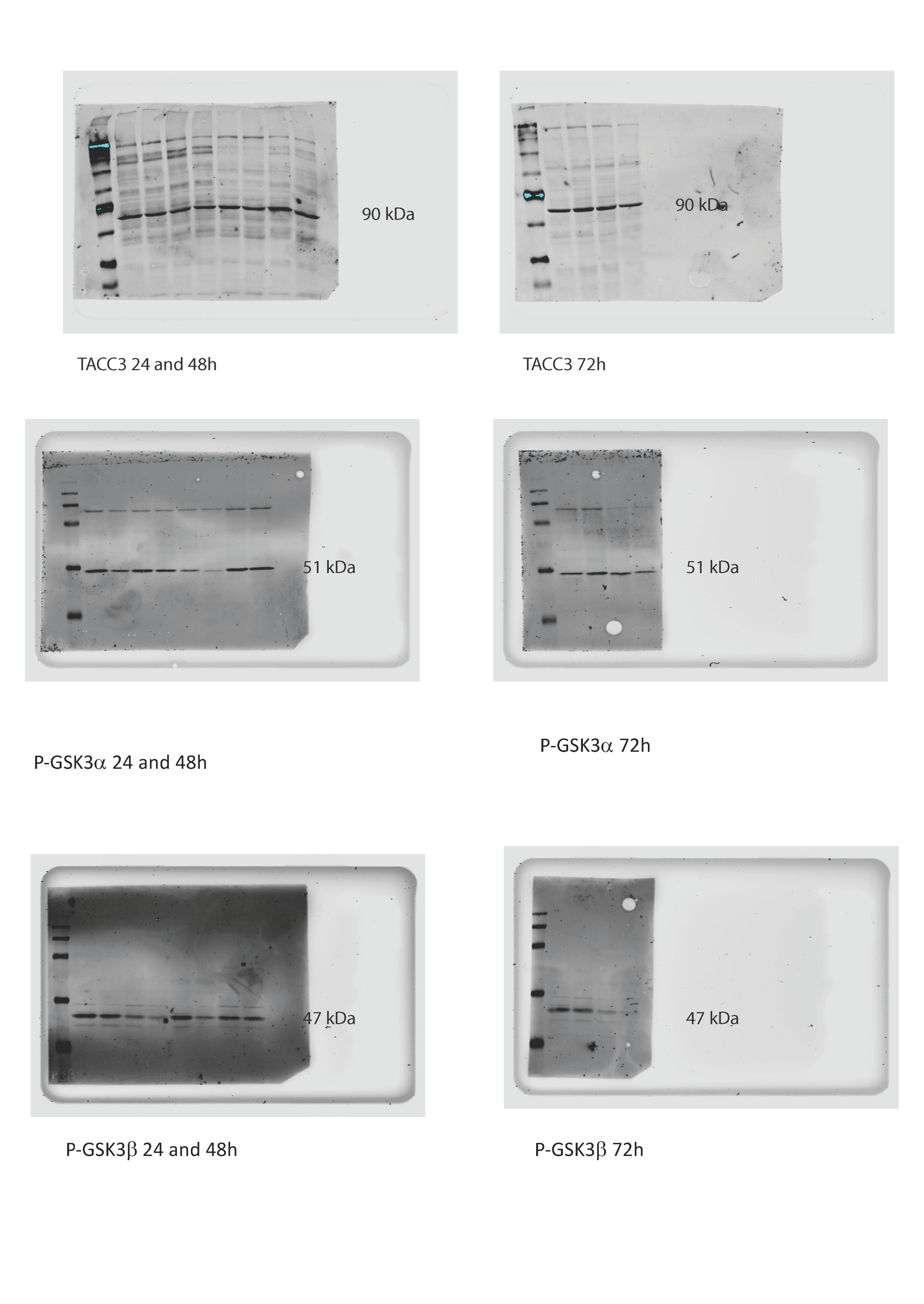

Supplement: Supplementary file 1 [file cancers-13-05939-s001.zip › Supplemental figures and legends/Supplemental figure 2C 241121.png]

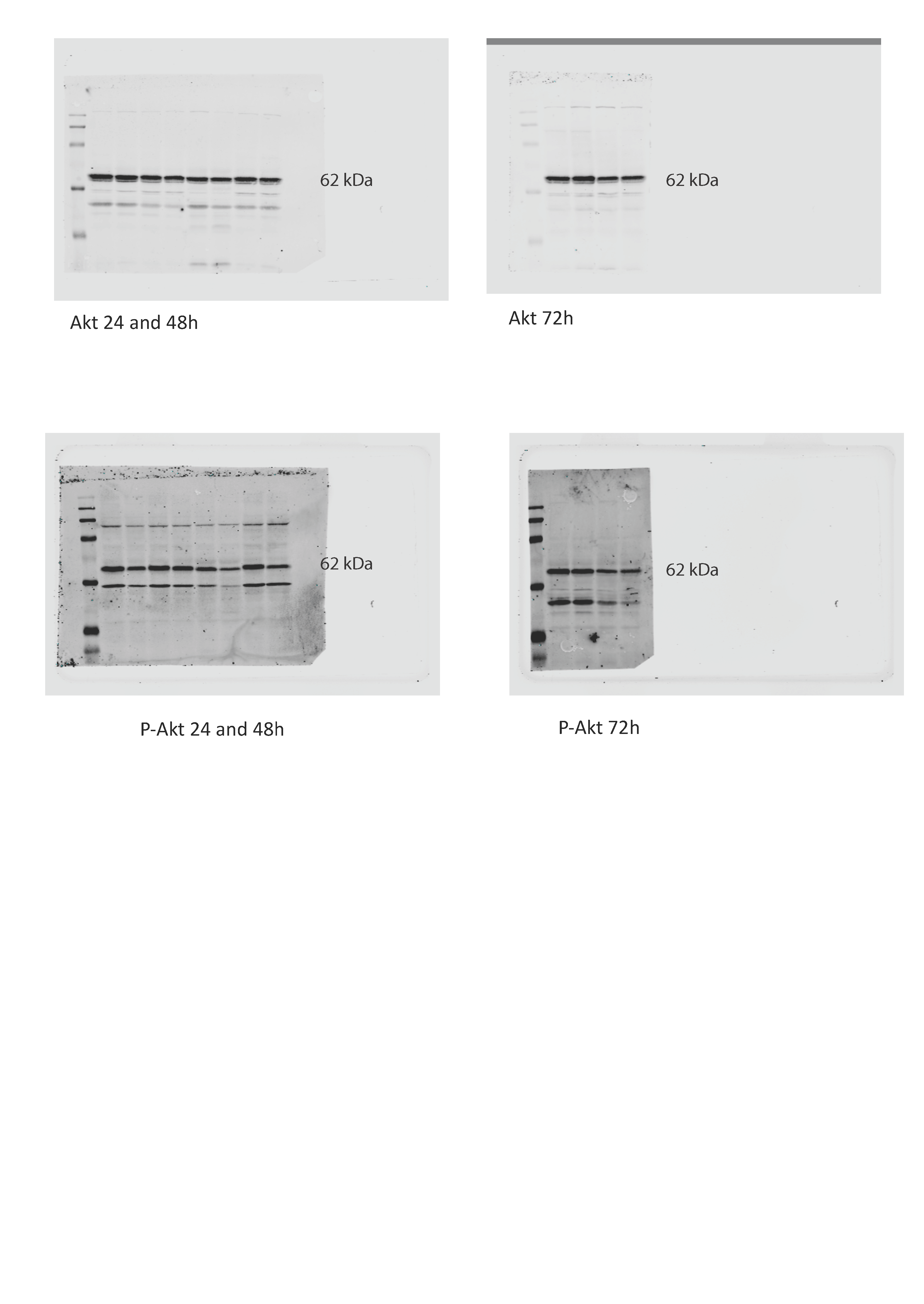

Supplement: Supplementary file 1 [file cancers-13-05939-s001.zip › Supplemental figures and legends/Supplemental figure 2D.png]

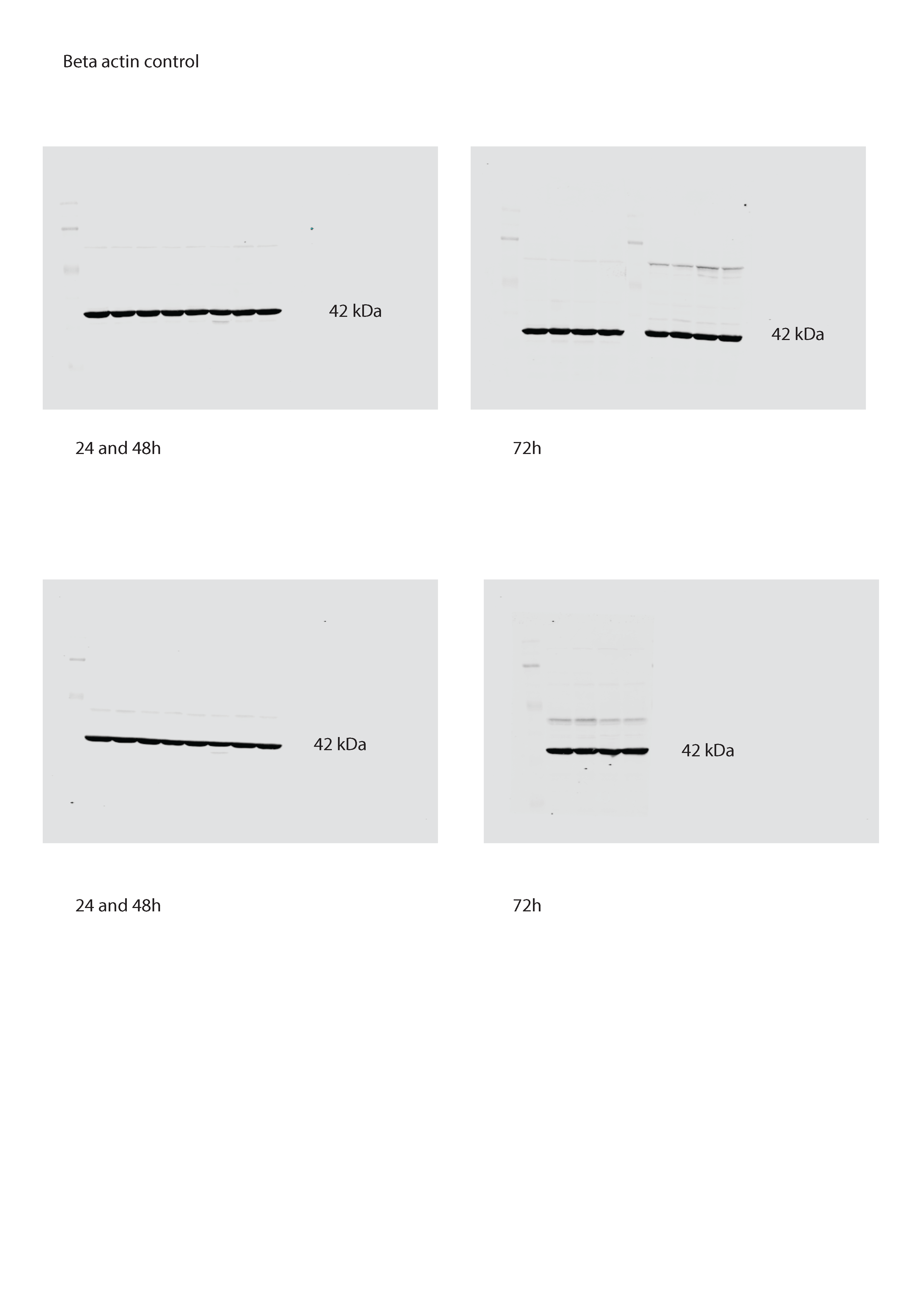

Supplement: Supplementary file 1 [file cancers-13-05939-s001.zip › Supplemental figures and legends/Supplemental figure 2E 241121.png]

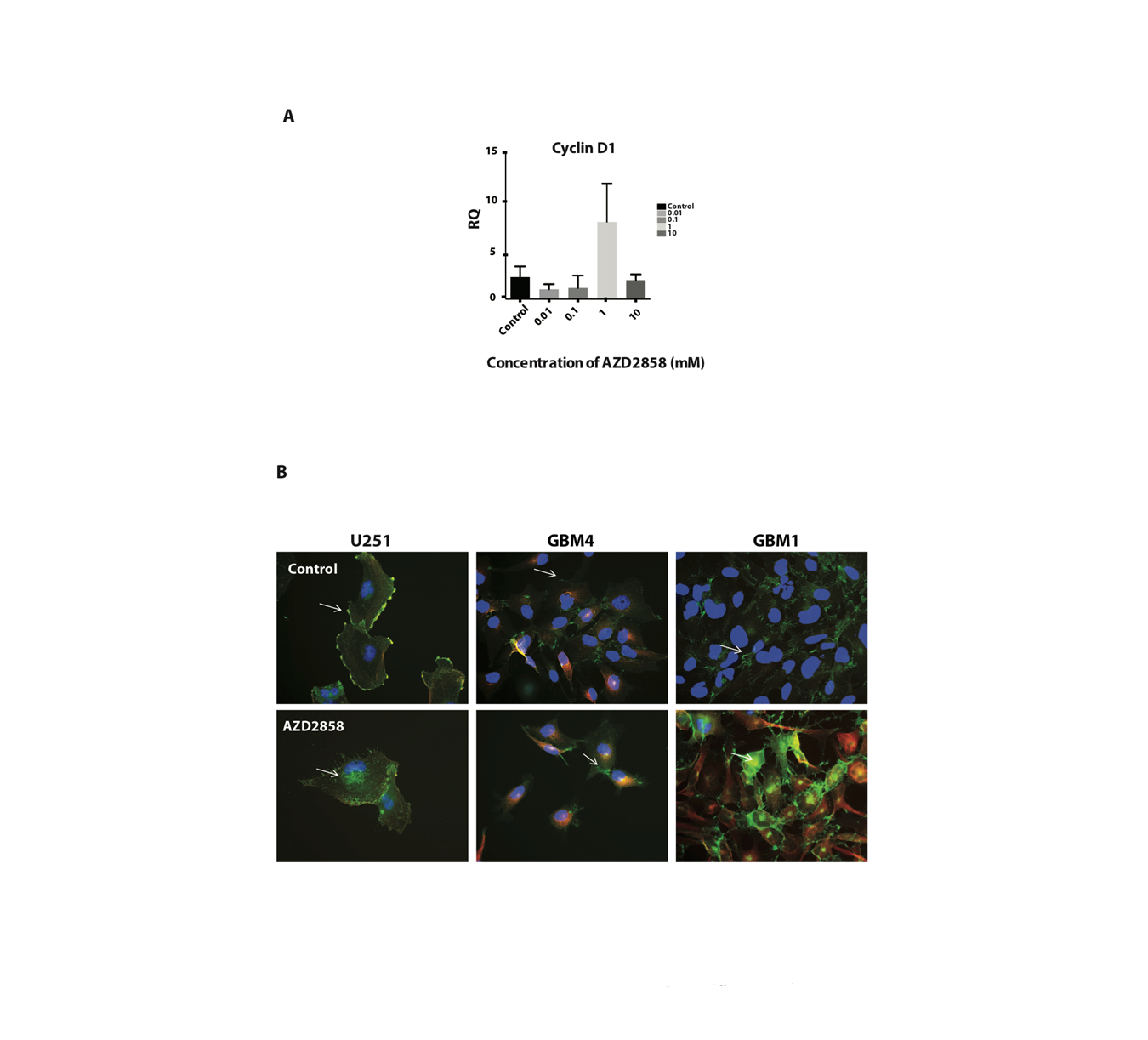

Supplement: Supplementary file 1 [file cancers-13-05939-s001.zip › Supplemental figures and legends/Supplemental figure 3 241121.png]

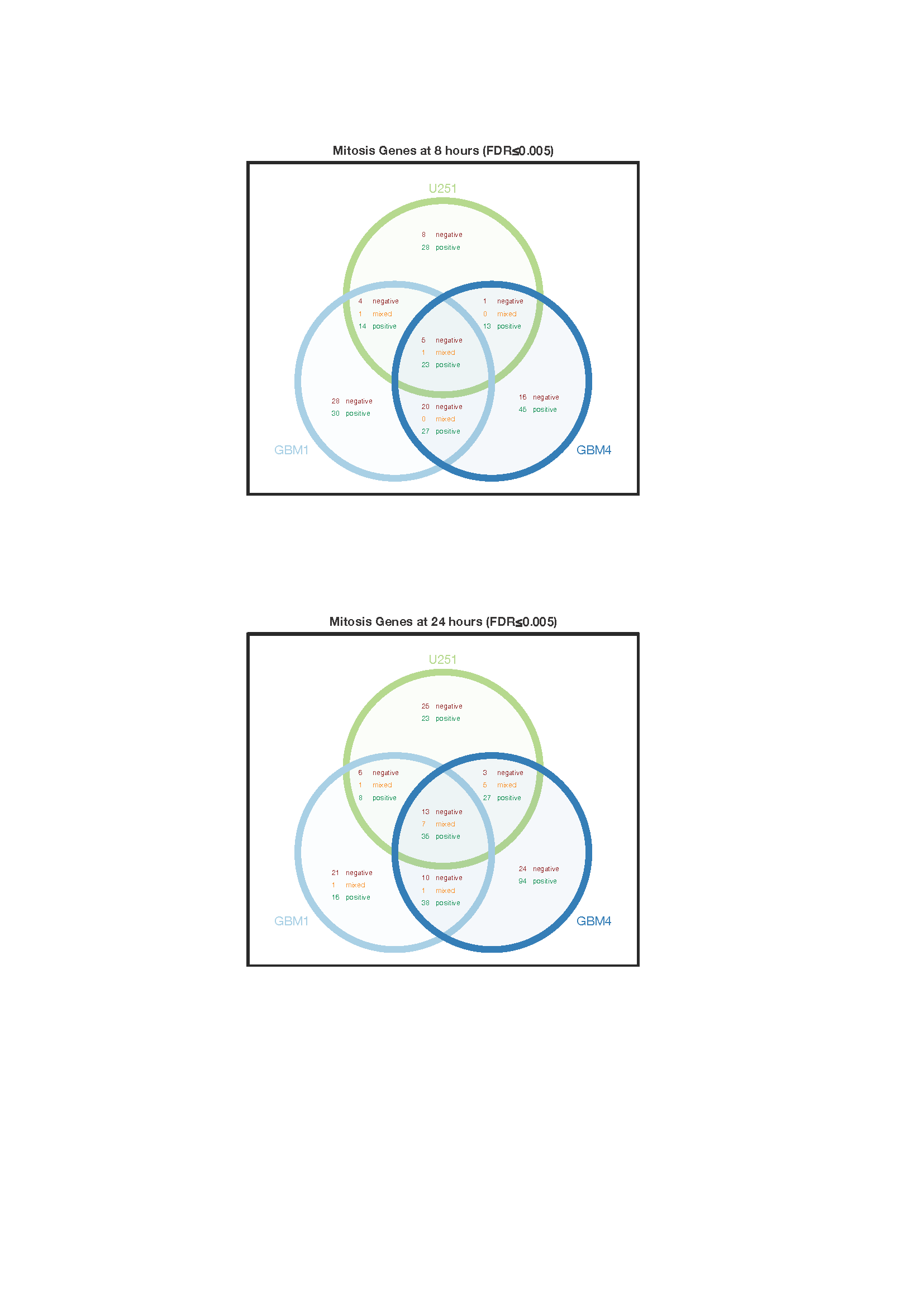

Supplement: Supplementary file 1 [file cancers-13-05939-s001.zip › Supplemental figures and legends/Supplemental figure 4 241121.png]

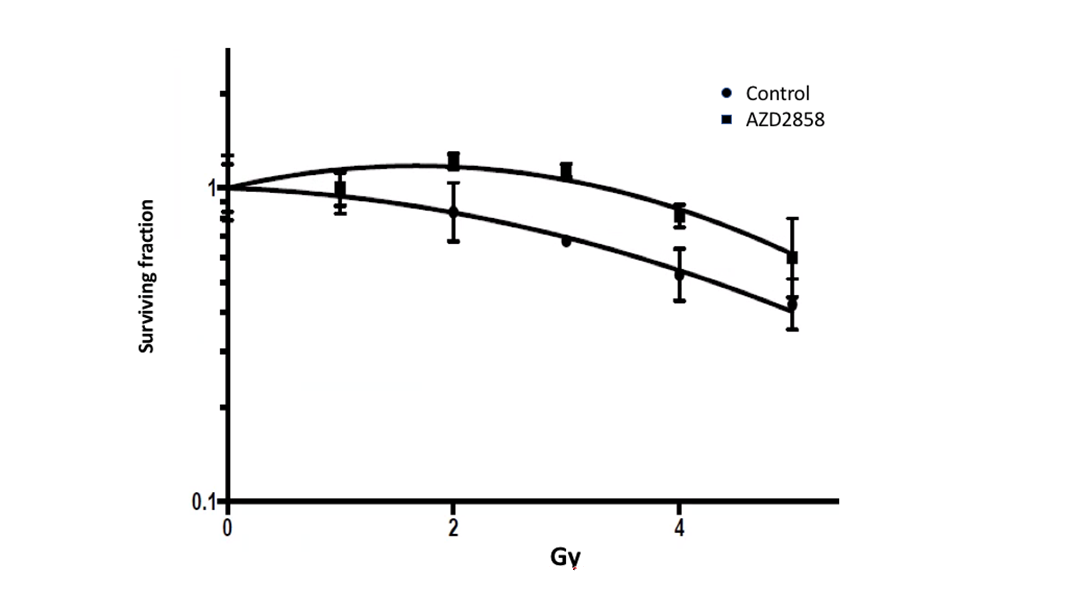

Supplement: Supplementary file 1 [file cancers-13-05939-s001.zip › Supplemental figures and legends/Supplemental figure 5.png]
